# CACA Guidelines for Holistic Integrative Management of Breast Cancer

**DOI:** 10.1007/s44178-022-00007-8

**Published:** 2022-07-05

**Authors:** Jiong Wu, Daiming Fan, Zhimin Shao, Binghe Xu, Guosheng Ren, Zefei Jiang, Yongsheng Wang, Feng Jin, Jin Zhang, Qingyuan Zhang, Fei Ma, Jinli Ma, Zhonghua Wang, Shusen Wang, Xiaojia Wang, Shu Wang, Haibo Wang, Tao Wang, Xiang Wang, Jing Wang, Jia Wang, Biyun Wang, Li Fu, Hongyuan Li, Yehui Shi, Lu Gan, Yunjiang Liu, Jian Liu, Zhenzhen Liu, Qiang Liu, Qiang Sun, Wenwu Cheng, Keda Yu, Zhongsheng Tong, Xinhong Wu, Chuangui Song, Jianguo Zhang, Jian Zhang, Junjie Li, Bin Li, Man Li, Huiping Li, Wentao Yang, Hongjian Yang, Benlong Yang, Hong Bu, Juping Shen, Zhenzhou Shen, Yiding Chen, Ceshi Chen, Da Pang, Zhimin Fan, Ying Zheng, Xiaoli Yu, Guangyu Liu, Xichun Hu, Yiqun Ling, Jinhai Tang, Yongmei Yin, Cuizhi Geng, Peng Yuan, Yajia Gu, Cai Chang, Xuchen Cao, Yuan Sheng, Yuanxi Huang, Jian Huang, Weijun Peng, Xiaohua Zeng, Yuntao Xie, Ning Liao, Committee of Breast Cancer Society, Chinese Anti-Cancer Association, Fan Daiming, Shao Zhimin, Xu Binghe, Ren Guosheng, Wu Jiong, Jiang Zefei, Wang Yongsheng, Jin Feng, Zhang Jin, Zhang Qingyuan, Ma Fei, Ma Jinli, Wang Zhonghua, Wang Yongsheng, Wang Shusen, Wang Xiaojia, Wang Shu, Wang Haibo, Wang Tao, Wang Xiang, Wang Jing, Wang Jia, Wang Biyun, Fu Li, Li Hongyuan, Shi Yehui, Gan Lu, Ren Guosheng, Liu Yunjiang, Liu Jian, Liu Zhenzhen, Liu Qiang, Cheng Wenwu, Jiang Zefei, Yu Keda, Tong Zhongsheng, Wu Xinhong, Song Chuangui, Zhang Jianguo, Zhang Jian, Zhang Qingyuan, Zhang Jin, Li Junjie, Li Bin, Li Man, Li Huiping, Yang Wentao, Yang Hongjian, Yang Benlong, Bu Hong, Shen Juping, Shen Zhenzhou, Shao Zhimin, Chen Yiding, Chen Ceshi, Pang Da, Fan Zhimin, Zheng Ying, Jin Feng, Yu Xiaoli, Liu Guangyu, Hu Xichun, Ling Yiqun, Tang Jinhai, Xu Binghe, Yin Yongmei, Geng Cuizhi, Geng Cuizhi, Geng Cuizhi, Yuan Peng, Gu Yajia, Chang Cai, Cao Xuchen, Sheng Yuan, Huang Yuanxi, Huang Jian, Huang Jian, Peng Weijun, Zeng Xiaohua, Xie Yuntao, Liao Ning

**Affiliations:** 1 Key Laboratory of Breast Cancer in Shanghai, Department of Breast Surgery, Fudan University Shanghai Cancer Center, Shanghai Medical College of Fudan University, Shanghai, China; 2 Department of Oncology, Shanghai Medical College of Fudan University, Shanghai, China; 3 Department of Gastroenterology, Beijing Chaoyang Hospital, Capital Medical University, Beijing, China; 4 State key Laboratory of Cancer Biology, National Clinical Research Center for Digestive Diseases and Xijing Hospital of Digestive Diseases, Fourth Military Medical University, Xi’an, China; 5 National Cancer Center/Cancer Hospital, Chinese Academy of Medical Sciences and Peking Union Medical College, Beijing, China; 6 Department of Endocrine and Breast Surgery, The First Affiliated Hospital of Chongqing Medical University, Yuzhong, Chongqing China; 7 Medicine-Oncology, The Affiliated Hospital of Military Medical Sciences (The 307th Hospital of Chinese People’s Liberation Army), Beijing, China; 8 Breast Disease Center, Shandong Cancer Hospital, Jinan, China; 9 Department of Breast Surgery, The First Affiliated Hospital of China Medical University, Shenyang, China; 10 The 3rd Department of Breast Cancer, China Tianjin Breast Cancer Prevention, Treatment and Research Center, Tianjin Medical University Cancer Institute and Hospital, National Clinical Research Center of Cancer, Tianjin, China; 11 Harbin Medical University Cancer Hospital, Harbin, China; 12 National Cancer Center/Cancer Hospital, Chinese Academy of Medical Sciences and Peking Union Medical College, Beijing, China; 13 Department of Radiation Oncology, Fudan University Shanghai Cancer Center, Shanghai Medical College of Fudan University, Shanghai, China; 14 Sun Yat-Sen University Cancer Center, Guangzhou, China; 15 Zhejiang Cancer Hospital, Hangzhou, China; 16 Breast Center, Peking University People’s Hospital, Beijing, China; 17 Department of Breast Center, The Affiliated Hospital of Qingdao University, Shandong, China; 18 Department of Oncology, Fifth Medical Center of Chinese PLA General Hospital, Beijing, China; 19 Department of Breast Surgical Oncology, National Cancer Center/National Clinical Research Center for Cancer/Cancer Hospital, Chinese Academy of Medical Sciences and Peking Union Medical College, Beijing, China; 20 Department of Breast Surgery, Institute of Breast Disease, The Second Hospital of Dalian Medical University, Dalian, Liaoning China; 21 Department of Medical Oncology, Fudan University Shanghai Cancer Centre, Shanghai, China; 22 Department of Breast Cancer Pathology and Research Laboratory, Tianjin Medical University Cancer Institute and Hospital, Tianjin, China; 23 Key Laboratory of Breast Cancer Prevention and Therapy, Tianjin Medical University, Ministry of Education, Tianjin, China; 24 State Key Laboratory of Breast Cancer Research, Tianjin, China; 25 Department of the Endocrine and Breast Surgery, The First Affiliated Hospital of Chongqing Medical University, Chongqing Medical University, Chongqing, China; 26 Department of Breast Oncology, Tianjin Medical University Cancer Institute and Hospital, National Clinical Research Center for Cancer, Tianjin, China; 27 Key Laboratory of Cancer Prevention and Therapy, Tianjin, China; 28 Tianjin’s Clinical Research Center for Cancer, Tianjin, China; 29 Department of Oncology, The First Affiliated Hospital of Chongqing Medical University, Chongqing, China; 30 Breast Center, The Fourth Hospital of Hebei Medical University, Shijiazhuang, China; 31 Department of Breast Surgery, Affiliated Hangzhou First People’s Hospital, Zhejiang University, School of Medicine, Hangzhou, Zhejiang China; 32 Department of Breast Disease, Henan Breast Cancer Center, Affiliated Cancer Hospital of Zhengzhou University & Henan Cancer Hospital, Zhengzhou, China; 33 Guangdong Provincial Key Laboratory of Malignant Tumor Epigenetics and Gene Regulation, Sun Yat-Sen Memorial Hospital, Sun Yat-Sen University, Guangzhou, China; 34 Breast Tumor Center, Sun Yat-Sen Memorial Hospital, Sun Yat-Sen University, Guangzhou, China; 35 Department of Breast Surgery, Peking Union Medical College Hospital, Peking Union Medical College, Chinese Academy of Medical Sciences, Beijing, China; 36 Department of Integrated Therapy, Fudan University Shanghai Cancer Center, Shanghai, China; 37 Department of Medical Oncology, Tianjin Medical University Cancer Institute and Hospital, Tianjin, China; 38 Hubei Provincial Clinical Research Center for Breast Cancer, Department of Breast Surgery, Hubei Cancer Hospital, Tongji Medical College, Huazhong University of Science and Technology, Wuhan, China; 39 Department of Breast Surgery, Fujian Medical University Union Hospital, Fuzhou, China; 40 Department of Breast Surgery, The Second Affiliated Hospital of Harbin Medical University, Harbin, Heilongjiang China; 41 Department of Nurse, Cancer Institute and Cancer Center, Fudan University, Shanghai, China; 42 The Second Affiliated Hospital of Dalian Medical University; Institute of Cancer Stem Cell, Dalian Medical University, Dalian, China; 43 Key Laboratory of Carcinogenesis and Translational Research (Ministry of Education/Beijing), Department of Breast Oncology, Peking University Cancer Hospital & Institute, Beijing, China; 44 Department of Pathology, Fudan University Shanghai Cancer Center, Shanghai, China; 45 Department of Breast Surgery, Zhejiang Cancer Hospital, Hangzhou, Zhejiang China; 46 Laboratory of Pathology, Key Laboratory of Transplant Engineering and Immunology, NHC, West China Hospital, Sichuan University, Chengdu, China; 47 Department of Pathology, West China Hospital, Sichuan University, Chengdu, China; 48 Department of Breast Surgery, The Second Affiliated Hospital, Zhejiang University School of Medicine, Hangzhou, China; 49 The Key Laboratory of Cancer Prevention and Intervention, China National Ministry of Education, Zhejiang University School of Medicine, Hangzhou, China; 50 Key Laboratory of Animal Models and Human Disease Mechanisms of the Chinese Academy of Sciences and Yunnan Province, Kunming Institute of Zoology, Chinese Academy of Sciences, Kunming, China; 51 KIZ-CUHK Joint Laboratory of Bioresources and Molecular Research in Common Diseases, Kunming Institute of Zoology, Chinese Academy of Sciences, Kunming, China; 52 Institute of Translation Medicine, Shenzhen Second People’s Hospital, The First Affiliated Hospital of Shenzhen University, Shenzhen, China; 53 Department of Breast Surgery, Harbin Medical University Cancer Hospital, Harbin, China; 54 Department of Breast Surgery, The First Bethune Hospital of Jilin University, Changchun, Jilin China; 55 Department of Cancer Prevention, Fudan University Shanghai Cancer Center, Shanghai, China; 56 Department of Radiotherapy, Fudan University Shanghai Cancer Center, Shanghai, China; 57 Department of Nutrition, Fudan University Shanghai Cancer Center, Shanghai, China; 58 Department of Breast Cancer, Jiangsu Province Hospital, Nanjing, China; 59 Department of Breast Surgery, Fourth Hospital of Hebei Medical University, Shijiazhuang, China; 60 National Cancer Center, National Clinical Research Center for Cancer, Cancer Hospital, Chinese Academy of Medical Sciences and Peking Union Medical College, Beijing, China; 61 Department of Radiology, Fudan University Shanghai Cancer Center, Shanghai, China; 62 Department of Medical Ultrasound, Fudan University Shanghai Cancer Center, Shanghai, China; 63 The First Department of Breast Cancer, Tianjin Medical University Cancer Institute and Hospital, National Clinical Research Center for Cancer, Tianjin, China; 64 Department of Breast Surgery, Changhai Hospital, The Second Military Medical University, Shanghai, China; 65 Department of Breast Surgery, Harbin Medical University Cancer Hospital, Harbin, China; 66 Department of Breast Surgery, Key Laboratory of Tumor Microenvironment and Immune Therapy of Zhejiang Province, Cancer Research Institute of Zhejiang University, The Second Affiliated Hospital, Zhejiang University School of Medicine, Zhejiang, China; 67 Breast Center, Chongqing Cancer Hospital, Chongqing University, Chongqing, China; 68 Key Laboratory of Carcinogenesis and Translational Research (Ministry of Education/Beijing), Breast Center, Peking University Cancer Hospital & Institute, Beijing, China; 69 Department of Breast Cancer, Cancer Center, Guangdong Provincial People’s Hospital, Guangdong Academy of Medical Sciences, Guangzhou, Guangdong China

**Keywords:** Epidemiology of breast cancer, Breast cancer screening, Breast cancer diagnosis, Early breast cancer treatment, Advanced breast cancer treatment, Traditional Chinese medicine treatment of breast cancer

## Abstract

**Purpose:**

Breast cancer is now the most common malignant tumor worldwide. About one-fourth of female cancer patients all over the world suffer from breast cancer. And about one in six female cancer deaths worldwide is caused by breast cancer. In terms of absolute numbers of cases and deaths, China ranks first in the world. The CACA Guidelines for Holistic Integrative Management of Breast Cancer were edited to help improve the diagnosis and comprehensive treatment in China.

**Methods:**

The Grading of Recommendations Assessment, Development and Evaluation (GRADE) was used to classify evidence and consensus.

**Results:**

The CACA Guidelines for Holistic Integrative Management of Breast Cancer include the epidemiology of breast cancer, breast cancer screening, breast cancer diagnosis, early breast cancer treatment, advanced breast cancer treatment, follow-up, rehabilitation, and traditional Chinese medicine treatment of breast cancer patients.

**Conclusion:**

We to standardize the diagnosis and treatment of breast cancer in China through the formulation of the CACA Guidelines.

## Part 1: Epidemiology of Breast Cancer

### Breast cancer prevalence and mortality

Breast cancer is the most common malignant tumor in the world. It ranks first among cancers in women in 159 countries out of 185 countries and is also the most common cause of death among women in 110 countries. According to the latest estimates from International Agency for Research on Cancer (IARC), World Health Organization (WHO) in 2020, there are 2.3 million new cases of breast cancer each year, accounting for 11.7% of all cancer cases worldwide, and 685,000 women die from  breast cancer annually, accounting for 6.9% of all cancer-related deaths globally, making breast cancer the fifth-leading cause of cancer-related deaths. Among female cancer patients all over the world, one-fourth suffer from breast cancer, and one-sixth of women cancer death worldwide is caused by of breast cancer [1].

In terms of breast cancer prevalence, the global geographic distribution varies profoundly. In developed countries with high level of industrialization, the incidence rate of breast cancer is higher, which is mainly related to differences in genetic factors, lifestyles, and environmental exposure factors.

The global distribution of breast cancer mortality is completely different from the incidence. In areas with lower incidence rates, such as in Africa, the standardized mortality rate is higher, mainly because cancer mortality is affected not only by the incidence rate but also by the level of clinical diagnosis, treatment, and rehabilitation [1].

### Breast cancer incidence and mortality of Chinese women

Breast cancer is one of the most common malignancies in Chinese women. The incidence and mortality of breast cancer in Chinese women appear to be lower than global prevalence. According to IARC estimates, the standardized incidence and mortality of breast cancer among Chinese women in 2020 are 39.1/100,000 and 10/100,000, respectively, which are both lower than the world’s averages. Nonetheless, given its large population, China still ranks first in the world for absolute numbers of cases and deaths [2].

According to data from the National Central Cancer Registry of China, in 2015 there were approximately 304,000 new female breast cancer cases nationwide, with an incidence rate of 45.29/100,000, and a world-standardized rate of 29.56/100,000, ranking first among female cancer cases. In 2015, there were approximately 70,000 women died of breast cancer in China, with a crude death rate of 10.5/100,000 and a world-standardized rate of 6.48/100,000, ranking 6th of all female deaths. The morbidity and mortality of breast cancer in urban areas were higher than those in rural areas [2–4].

### Characteristics of breast cancer disease in Chinese women

#### Age distribution

The age-specific incidence rate of breast cancer in Chinese women increases with age. Chinese breast cancer patients have an early onset, and, the incidence rate rapidly rises with age after 30, reaching a peak at 55, and remains high after this [2, 3].

#### Distribution of histological types

Breast cancer originates from mammary ducts at all levels and acinar epithelium of the breast, 95% of which are malignant epithelial tumor. According to the classification of the WHO’s International Classification of Diseases for Oncology, third edition (ICD-O-3) [5], the tumor registration data in Shanghai, China, shows that invasive ductal carcinoma accounts for 70% of breast cancer cases in Chinese women [4].

#### Distribution of breast cancer stages

According to China’s multicenter clinical data, at the time of diagnosis, only 15.7% of breast cancer patients in some urban and rural areas in China are at stage I. The Shanghai population tumor registration data and the Shanghai breast cancer cohort study are population-based. The data show that stage I breast cancer cases only account for 25% to 35% of the total cases. To a certain extent, it may also represent the situation of other larger cities in China, where better medical resources are provided [4].

#### Molecular subtyping of breast cancer

Several large studies that analyzed the distribution of molecular subtypes of female breast cancer in China show that in Chinese female breast cancer cases, the proportions of luminal A, luminal B, and triple-negative breast cancer cases are 40–70%, 10–20%, and 15–20%, respectively [6, 7].

### Risk factors for breast cancer

The etiology and pathogenesis of breast cancer are very complex. Breast cancer is caused by multiple interacting factors,  such as genetics, lifestyle, and environmental exposure. Genetic mutations in breast cancer susceptibility genes increase the risk of breast cancer. Reproductive factors, including late menarche, early menopause, parity, early primiparous age, and breastfeeding, can lower the overall risk of breast cancer. Family history of breast cancer, history of benign breast proliferative disease, dense breast tissue, radiation exposure, alcohol consumption, low physical activity, premenopausal thinness, postmenopausal obesity, recent use of postmenopausal hormone therapy (especially estrogen combined with progesterone), and recent use of oral contraceptives are associated with higher overall risk of breast cancer [8].

## Part 2: Breast Cancer Screening

### Definition, objectives, and types of breast cancer screening

1. Tumor screening, also known as general screening, is a cancer prevention measure for asymptomatic population, while medical examination of symptomatic individuals is called diagnosis [8].
2. Breast cancer screening refers to the screening on asymptomatic women through effective, simple, and economical breast examination measures for early detection, early diagnosis, and early treatment, its ultimate goal is to reduce the mortality rate of breast cancer in the population [8].
3. Breast cancer screening can be categorized into two types: opportunistic screening and mass screening. Opportunistic screening refers to breast screening provided by healthcare institutions for women of appropriate age who seek for medical assistance on various conditions, or those voluntarily visiting health care institutions that provide breast screening services. Mass screening refers to breast screening services for women of appropriate age provided by community or institutional entities with the aid of equipment, technology, and personnel from health care institutions in an organized manner [8, 9].

### Starting and ending ages for women’s breast cancer screening

1. Most guidelines recommend to start breast cancer screening at age 40. In a systematic review of foreign countries that included eight randomized controlled trials, in which women under 50 (*n* = 347,851) were compared between two groups, i.e., with and without mammography screening, it was found that the relative risk of breast cancer–induced mortality of mammography screening group was 0.88 (95% confidence interval (CI): 0.76 to 1.02, moderate-quality evidence). The peak age of breast cancer in Chinese women is 45-54 years, approximately 10 years earlier than that of women in European and American countries. Therefore, this guideline recommends that the starting age of breast cancer screening for general risk populations to be 40 years (recommendation level 2), and women aged 45-49 years should use mammography for breast cancer screening (recommendation level 1). For people at high risk for breast cancer, the starting screening age can be arranged before 40 advanced to before age 40 years [8, 9].
2. Regarding the ending age of breast cancer imaging screening, most foreign guidelines on mass screening recommend 65-70 years as the last age for screening (low-quality evidence). The guideline expert group recommends that women aged 70-74 years can opt in or out of mammography for breast cancer screening (moderate-quality evidence, recommendation level 2). However, the incidence of breast cancer in elderly women is still high, so this guideline recommends that the individual's physical health, life expectancy, and various comorbidities should put into consideration to decide if screening should be stopped. For those with many complications and limited life expectancy, breast cancer screening can be appropriately reduced (recommendation level 2). Thus, opportunistic screening can be considered for women over 70 [8, 9].

### Measures for Breast Cancer Screening

#### Mammography examination

1. The effectiveness of mammography examination in reducing breast cancer mortality in women over 40 is recognized by most scholars in China and abroad (moderate-quality evidence). One systematic review included 249,930 women in six randomized controlled trials to compare the differences between patients with and without mammography screening. The short-term case accumulation method was used for statistical analysis, and the average screening duration was 6.3 years. The results of the meta-analysis showed that compared with non-mammography screening group, the relative risk of death from breast cancer in mammography screening group was 0.77 (95% CI: 0.66 to 0.90, high-quality evidence). It also showed that compared with the non-mammography screening group, the mammography screening group had a lower risk of IIA breast cancer (very low-quality evidence) and lower risk of stage III or above breast cancer or ≥ 40-mm breast cancer (low-quality evidence), though it had the same risk of death from all causes or other causes (low-quality evidence) [8].
2. It is recommended that in routine mammography, each breast should be examined under two positions, i.e., the craniocaudal (CC) position and the mediolateral oblique (MLO) position [8].
3. Mammography images should be independently read by more than two professional radiologists.
4. Mammography screening has high diagnostic accuracy in Asian women over 50 but is not accurate enough for diagnosis in women under 40 or women with dense breast tissue. Mammography is not recommended for women under 40 who have no clear high-risk factors for breast cancer or have not shown any abnormality on physical examination (low-quality evidence, recommendation level 1) [8].
5. The radiation dose of conventional mammography is low and does not pose any harm to women’s health, but normal women should not be subjected to repeated mammography examinations in a short period of time. Instead of once a year, it is recommended that women aged 40-49 years undergo mammography screening once every 2 years (very low-quality evidence, recommendation level 2); instead of once every 3 years, it is recommended that women aged 40-49 years undergo mammography screening once a year (very low-quality evidence, recommendation level 2); instead of once every 3 years, it is recommended that women aged 40-49 years undergo mammography screening once every 2 years (very low-quality evidence, recommendation level 2). It is more advantageous for women aged 50-69 years to receive mammography screening once every 2 years than every 3 years, and it is more advantageous for them to receive it once a year than once every 3 years (low-quality evidence, recommendation level 1) [8, 9].

#### Breast ultrasonography examination

There is much evidence suggesting that combining mammography with breast ultrasonography has higher screening sensitivity than mammography alone, especially for women with dense breast tissue (type c or d) as indicated by mammography screening. Therefore, breast ultrasonography can be recommended as an effective supplement to mammography screening (moderate-quality evidence, recommendation level 2). A systematic review and meta-analysis of 26 studies found that the sensitivity of ultrasound as a screening method for breast cancer was 80.1% (95% CI: 0.722 to 0.863), and the specificity was 88.4% (95% CI: 0.798 to 0.936). If only data from low- and middle-income countries were included, then the sensitivity and specificity rose to 89.2% and 99.1%, respectively. The findings of the studies support the use of ultrasound as a screening method for breast cancer in low-income countries or areas with relatively limited resources and for women with unreliable mammography results, such as those with dense breast tissue (moderate-quality evidence). However, in mass screenings, the addition of ultrasonography will inevitably increase screening cost, and the cost-effectiveness of breast ultrasonography will thus be weakened. In addition, the effectiveness of breast ultrasonography alone as a screening measure has not been fully confirmed by evidence [8, 9].

#### Breast clinical examination

There is currently no evidence that clinical breast examination alone as a method of breast cancer screening can improve the accuracy of early breast cancer diagnosis and reduce mortality. However, it can still be used as an option in areas with underdeveloped economies, limited equipment, and low awareness of the disease among women (very low-quality evidence, recommendation level 2) [9].

#### Breast self-examination

1. Breast self-examination can neither increase the early detection rate of breast cancer nor reduce the mortality rate (very low-quality evidence) [8].
2. Since breast self-examination methods can improve women’s awareness of cancer prevention, primary medical workers are encouraged to teach women the method and encourage them to use it monthly. It is recommended that premenopausal women perform breast self-examination 7 to 14 days after menstruation (very low-quality evidence, recommended Level 2) [8].

#### Breast magnetic resonance imaging (MRI) examination

1. MRI can be a supplementary measure for suspected cases found by mammography, clinical breast examination, or breast ultrasonography [9].
2. It can be combined with mammography for breast cancer screening for *BRCA1/2* gene mutation carriers (very low-quality evidence, recommendation level 2) [9].

#### Other examinations

The current evidence does not support near-infrared scanning, radionuclide scanning, catheter lavage, or blood oxygen testing as effective breast cancer screening methods (very low-quality evidence, recommendation level 2) [8, 9].

### Breast cancer screening guideline for women at general risk

Women at general risk of breast cancer refer to all women except those at high risk of breast cancer (see section 5.1 for definition) [8, 9].

#### Women aged 20–39 years

Breast cancer screening is not recommended for this age group (low-quality evidence, recommendation level 1).

#### Women aged 40–70 years

1. Suitable for opportunistic screening and mass screening.
2. Undergo mammography once every 1 or 2 years. For those with dense breast tissue (with type c or type d glands revealed by mammography), the combination of mammography and B-mode ultrasonography is recommended (moderate-quality evidence, recommendation level 1).

#### Women over 70

1. Suitable for opportunistic screening.
2. Undergo mammography once every 1 to 2 years (very low-quality evidence, recommendation level 2).

### Recommendations for screening of high-risk groups of breast cancer

It is recommended that people at high risk of breast cancer be screened early (before the age of 40), at a frequency of once a year. In addition to mammography, imaging methods such as B-mode ultrasonography and MRI can also be employed (moderate-quality evidence, recommendation level 2). In a randomized controlled study in China, in which ultrasonography, mammography, and the combination of ultrasonography and mammography were used as preliminary screening methods for Chinese women aged 30-65 years at high risk of breast cancer, the results showed that as a preliminary screening method, ultrasonography shows higher sensitivity than and similar specificity to mammography (moderate-quality evidence) [9].

### Definition of high-risk group of breast cancer

Individuals meeting any of the following criteria are considered to be at high risk of breast cancer [9, 10].

1. Obvious genetic predisposition to breast cancer, for which the main criteria are as follows:
  a. First-degree relatives have a history of breast or ovarian cancer;
  b. Two or more second-degree relatives have been diagnosed with breast cancer before the age of 50;
  c. Two or more second-degree relatives have been diagnosed with ovarian cancer before the age of 50;
  d. At least one first-degree relative carries a known pathogenic genetic mutation of the *BRCA1/2* gene; or the woman carries a pathogenic genetic mutation of the *BRCA1/2* gene (see Table 8 in Appendix for individuals who require the *BRCA* genetic testing).
2. History of breast duct or lobular dysplasia or of lobular carcinoma *in situ* (LCIS).
3. Underwent chest radiotherapy before the age of 30.
4. After the assessment of the risk of breast cancer using the Gail model based on multiple risk factors such as age, race, age at menarche, age at primipara, personal breast disease history, family history of breast cancer, and number of breast biopsies, etc., the subject is considered high-risk if their 5-year risk of developing the disease is ≥1.67%.

Note: First-degree relatives refer to mother, daughters, and sisters; second-degree relatives refer to paternal aunts, maternal aunts, paternal grandmother, and maternal grandmother.

#### Recommended screening strategy and management for women at high risk of breast cancer (low-quality evidence, recommendation level 2)

1. Breast screening is recommended to be performed earlier than the designated starting age (< 40 years old).
2. Mammography once a year.
3. Breast ultrasonography once every 6 to 12 months.
4. Breast physical examination once every 6 to 12 months.
5. Combined contrast-enhanced breast MRI examination if necessary.

## Part 3: Breast Cancer Diagnosis

### Routine mammography examination and reporting specifications

#### Technical specifications of mammography

##### Preparations before mammography

Medical technicians should patiently explain the situation to the examinee so that the examinee is relaxed, understands the procedure, and cooperates with the examination.

##### Routine body position for mammography

Correctly positioning the body is a prerequisite for obtaining high-quality mammographic images. The bilateral mediolateral oblique (MLO) position and the craniocaudal (CC) position are common positions for mammography.

##### Supplementary body positions for mammography and mammogram techniques

If poor or incomplete breast parenchyma is revealed through the MLO position or the CC position, the following body positions can be used as supplementary positions according to the location of the lesion:

Level of evidence: high; level of recommendation: recommended.

#### Diagnostic report specifications

X-ray signs of abnormal manifestations such as intramammary lumps and calcifications should be described in reference to the Breast Imaging Reporting and Data System (BI-RADS) Classification Criteria of the American College of Radiology.

##### Lump

A contoured space-occupying lesion seen at two mutually perpendicular (or near-perpendicular) projections; if seen only at one projection, it should be described as "asymmetric" before it is determined to have the 3D space-occupying feature. The description of the lump should include three aspects: edge, shape, and density, and the signs of the lump edge are the most important for determining the lump’s nature.

1.2.1.1 Lump edge descriptions

① Distinct; ② obscured; ③ lobulated; ④ fuzzy; ⑤ stellate.

1.2.1.2 Lump shape descriptions

A lump can have a round, oval or irregular shape.

1.2.1.3 Lump density descriptions

Compared with the breast tissue of equal volume around the lump, a lump is categorized into four types: high density, equal density, low density (not fat-containing), and fat-containing density.

##### Calcification

The description of calcified lesions should include two aspects: type and distribution.

1.2.2.1 Type of calcification

It can be divided into typical benign calcifications and suspicious calcifications.

1. Benign calcifications have the following manifestations:

① Skin calcification; ② vascular calcification; ③ coarse or popcorn-like calcification; ④ large rod-like calcification; ⑤ round and point calcification; ⑥ rim calcification; ⑦ calcium milk-like calcification; ⑧ suture calcification; and ⑨ dystrophic calcification.

(2) Suspicious calcifications have the following manifestations:

① Amorphous calcification; ② coarse and heterogeneous calcification, probably malignant when distributed in individual clusters, with a positive predictive value (PPV) of malignancy of approximately 15% (BI-RADS category 4B); ③ fine pleomorphic calcifications (BI-RADS category 4B); and ④ fine-linear or fine-linear and branching calcifications (BI-RADS category 4C).

1.2.2.2 Distribution of calcification

① Diffuse distribution; ② regional distribution; ③ clustered distribution; ④ linear distribution; ⑤ segmental distribution.

1.2.3 Architectural distortion

1.2.4 Signs of symmetry

1.2.5 Intramammary lymph nodes

1.2.6 Skin lesions

1.2.7 Unilateral dilated duct

1.2.8 Combined signs

Combined signs include sunken skin, sunken nipple, retracted nipple, thickened skin, thickened trabecular structure, enlarged axillary lymph node, architectural distortion, and calcification.

Level of evidence: high; level of recommendation: recommended.

#### Localization of lesions

① A distinct 3D lesion must be present in the breast. For confirmation, the lesion needs to be seen in two projections, preferably in two mutually perpendicular projections. The following aspects must be clarified: ① on which side of the breast; ② location; ③ depth; ④ and distance from the nipple.

Level of evidence: high; level of recommendation: recommended.

#### Composition of mammogram report

The report should contain the patient’s medical history, objectives of the examination, imaging position, breast type, important imaging findings and the result of comparison with previous examinations, which are then followed by the assessment category and recommendations. The wording of the report should be concise and use standard terms from the latest approved lexicon.

##### Objectives of examination

The purpose of a mammogram (e.g., screening on asymptomatic women, recall examination after the screening, evaluation of clinical findings, or follow-up, etc.) should be concisely explained.

##### Breast type

The breast can be divided into four types: ① type a: fatty; ② type b: scattered fibroglandular; ③ type c: heterogeneously dense; ④ type d: dense.

##### Clearly describe any important findings

① Lumps; ② calcifications; ③ architectural distortion; ④ signs of asymmetry; ⑤ intramammary lymph nodes; ⑥ skin lesions; and ⑦ individually dilated ducts.

1.4.4 Compare with previous examinations

1.4.5 Assessment and classification

The BI-RADS classification is the most commonly used.

1.4.5.1 Assessment is incomplete

BI-RADS 0: Recall is required, with supplementation of other imaging examinations.

1.4.5.2 Assessment is complete: final classification

BI-RADS category 1: negative, and the likelihood of malignancy is 0%.
BI-RADS category 2: the likelihood of malignancy is 0%.
(3)BI-RADS category 3: the likelihood of malignancy of this type of lesion is 0–2%.
BI-RADS category 4: the likelihood of malignancy is 2–95%. It can be further subdivided into ① category 4A: the likelihood of malignancy is 2–10%; includes a group of lesions that are intervened by interventional methods but have a low likelihood of malignancy; ② category 4B: the likelihood of malignancy is 10–50%; ③ category 4C: higher suspicion of malignancy, but not yet a lesion of category 5, and the likelihood of malignancy is 50–95%.
BI-RADS category 5: highly suspected malignancy (almost certainly malignant), for which appropriate clinical measures should be taken. The likelihood of malignancy is ≥ 95%.
BI-RADS category 6: biopsy-proven malignancy. Active treatment measures should be taken.

Note: The formulation of this specification refers to the 5th edition of BI-RADS of American College of Radiology.

Level of evidence: high; level of recommendation: recommended.

### Breast ultrasonography and reporting specifications

#### Instruments for ultrasonography

Routine ultrasonography adopts a real-time linear-array high-frequency probe of the color Doppler ultrasound system. The probe frequency is set to 7.5–10.0 MHz, or to 10.0–15.0 MHz or higher if conditions permit.

Level of evidence: high; level of recommendation: recommended.

#### Methods of ultrasound examination

Pay attention to the completeness of the examination range to avoid missed inspections, and meanwhile, axillary lymph nodes should be examined. If necessary, lymph nodes above and below the clavicle as well as cervical lymph nodes should be examined.

Level of evidence: high; level of recommendation: recommended.

#### Procedure of ultrasound examination

##### Basic requirements

During the examination, a comprehensive conventional 2D ultrasound examination should be performed on the breast and surrounding tissues, and then a focused 2D ultrasound examination should be performed on any area where lesions are found. The examination items should include the measurements of the location, size, or range of the lesion as well as the border, edge, shape, interior and posterior echoes, and calcifications of the lesion and the changes in surrounding tissues (including architectural features such as skin, pectoral muscles, and ligaments). On the basis of 2D ultrasonography, axillary color and power Doppler ultrasonography should be employed to observe the direction and distribution of blood flow, and various blood flow parameters on the Doppler spectrum should be measured. Other techniques such as 3D ultrasound imaging, ultrasonic elastography, and contrast-enhanced ultrasound can be used to help improve the diagnosis if conditions permit.

Level of evidence: high; level of recommendation: recommended.

##### Image storage

The storage content of the image should include the patient's name, age, sex, and medical record number (outpatient number or hospitalization number, ultrasound registration number), as well as the equipment name and information on the inspection condition.

##### Report generation

The results of the above inspection items and the measured parameters should be described in detail in the ultrasound report. Finally, the ultrasound diagnostic results are presented by combining the various examination results.

Level of evidence: high; level of recommendation: recommended.

#### Specifications of ultrasound diagnostic report

To make ultrasound reports individualized but also standardized, the descriptive language of ultrasound reports should be defined first.

##### Echo mode of breast ultrasound

According to the intensity of the echo, it is categorized into weak echo, low echo, medium echo, high echo and strong echo.

##### Ultrasonographic manifestations of normal breast tissue

From superficial to deep, ultrasonographic images of normal breasts include ① skin; ② superficial fascia and subcutaneous fat; ③ mammary glands; ④ deep fascia; and ⑤ pectoral muscle and ribs.

##### Ultrasonographic manifestations of abnormal breast tissue

Breast abnormalities should be comprehensively observed in different sections to exclude normal tissues and architectures, such as fatty tissues and ribs. The ultrasonographic manifestations of focal lesions should be described in accordance with the following signs:

2.4.3.1 Lump

Shape, aspect ratio, border, edge, echo pattern, and posterior echo of the lesion.

2.4.3.2 Surrounding tissue

1. Edema and thickening of the skin and subcutaneous adipose layer.
2. Sunken, uneven skin.
3. Edema in surrounding tissue of the lesion.
4. Changes in architecturally distorted skin, superficial fascia layer, glandular layer, deep fascia layer, and pectoral muscle layer.
5. Cooper ligament changes.
6. Ductal changes.

2.4.3.3 Calcification

2.4.3.4 Vascular assessment

Level of evidence: high; level of recommendation: recommended.

##### Color Doppler ultrasonography

Color Doppler ultrasonography is used to examine the glandular tissue and blood vessels within the lesion. Except that of the resistance index (RI), the diagnostic significance of other parameters is still controversial. Generally, the RI of malignant lesions is > 0.70.

##### Other related techniques

2.4.5.1 3D ultrasound imaging

The most important role that 3D ultrasound imaging plays in breast lesion examination is the observation of the coronal surface of the lesion, rather than the 3D reconstruction of the lesion, which cannot be done through 2D ultrasound.

Level of evidence: intermediate; level of recommendation: recommended.

2.4.5.2 Ultrasonic elastography

Ultrasonic elastography examines the difference in elasticity of different tissues. It is generally believed that most of the tissues in malignant tumors are rigid. Due to the various settings of instruments made by different manufacturers, there is no unified diagnostic standard for ultrasonic elastography yet.

Level of evidence: intermediate; level of recommendation: recommended.

2.4.5.3 Contrast-enhanced ultrasound

Applications of contrast-enhanced ultrasound in the diagnosis of breast diseases are affected by factors such as the frequency of the probe, the contrast agent, and the growth of blood vessels in the lesion. There is no mature standard.

Level of evidence: intermediate; level of recommendation: recommended.

#### Classification of breast ultrasound assessment

The classification criteria of this guideline refer to the fifth edition of the BI-RADS Classification Criteria of American College of Radiology (2013), and the following classification criteria have been formulated according to the real-world situation in China.

##### Assessment is incomplete

BI-RADS category 0: needs further evaluation by other imaging examinations (e.g., mammography, MRI).

##### Evaluation is complete: classification

1. BI-RADS category 1: negative.
2. BI-RADS category 2: benign.
3. BI-RADS category 3: probably benign. Short-term (3-6 months) reexamination and adding other examinations are recommended.
4. BI-RADS category 4: suspicion of a the malignant lesion. The likelihood of malignancy of such lesions is 2% to 95%. They can be subdivided into subcategories 4A, 4B, and 4C, whose malignancy diagnosis accordance rates are 2–10%, 10–50% and 50–94%, respectively.
5. BI-RADS category 5: highly suggestive of malignancy, with a likelihood of malignancy of ≥ 95%. Active treatment should be initiated, including percutaneous biopsy (usually imaging-guided core-needle biopsy) or surgical treatment.
6. BI-RADS Category 6: malignancy has been confirmed by biopsy.

Level of evidence: high; level of recommendation: recommended.

#### Composition of breast ultrasound report

The wording of the report should be specific and concise, using unmodified terms. The report should include the following contents:

2.6.1 Patient information

2.6.2 Overall ultrasonographic description of bilateral breast tissue

2.6.3 Ultrasonographic description of significant abnormalities and lesions

2.6.3.1 Record of lesion

The general information should include the lesion’s side, location (with consistent and repeatable systematic positioning, such as clock positioning, skin distance from the nipple), and size (at least two diameters, and preferably three diameters if large).

2.6.3.2 Description of ultrasonographic image of the lesion

Lesions should be described one by one according to the content of the BI-RADS classification standards, including the shape, border, edge, interior and posterior echoes of the lesion, surrounding tissues, calcifications and blood flow in and around the lesion.

2.6.3.3 Conclusion

The conclusion section should contain whether the breasts are normal or abnormal, the physical properties of any detected lesions, and their corresponding diagnostic classifications and treatment recommendations (with the classification as the default content). An appropriate clinical diagnosis should be presented if possible.

2.6.3.4 Lesion image storage.

Level of evidence: high; level of recommendation: recommended.

## Routine breast MRI examination and reporting specifications

### Indications for breast MRI examination

#### Diagnosis of breast cancer

When mammography or ultrasonography finds a lesion but cannot determine its nature, further MRI examination can be considered.

Level of evidence: high; level of recommendation: recommended.

#### Breast cancer staging

Find multifocal and multicentric tumors, and evaluate the tumor's infiltration of the skin, pectoralis fascia, pectoralis major muscle, and chest wall.

Level of evidence: intermediate; level of recommendation: recommended.

#### Evaluation of effect of neoadjuvant therapy

MRI examinations before, during, and at the end of neoadjuvant treatment and before surgery can help evaluate the response of the lesions to treatment and allow a more accurate assessment of the range of residual lesions after treatment than conventional imaging techniques.

Level of evidence: high; level of recommendation: recommended.

#### Axillary lymph node metastasis, unknown primary focus

Breast MRI examinations can help find hidden cancer in the breast and determine its location and scope, to choose further treatments.

Level of evidence: high; level of recommendation: recommended.

#### Application before and after breast-conserving surgery

The application of MRI before breast-conserving surgery allows more accurate determination of the scope of lesions. In follow-ups after breast-conserving surgery, MRI is more conducive to identifying tumor recurrence and postoperative scars than conventional imaging techniques.

Level of evidence: high; level of recommendation: recommended.

#### Follow-up after mammoplasty

For women with breast prosthesis implantation, MRI can help evaluate the integrity of implanted prostheses and determine whether breast cancer has occurred.

Level of evidence: intermediate; level of recommendation: recommended.

#### Screening of high-risk groups

The age of breast cancer screening in high-risk groups is earlier than that in non-high-risk groups. MRI is helpful for evaluating the integrity of implanted prostheses and judging whether breast cancer exists.

Level of evidence: intermediate; level of recommendation: recommended.

#### MRI-guided puncture biopsy

MRI-guided puncture biopsy is only suitable for lesions discovered by MRI, and the abnormalities of target lesions cannot be confirmed through ultrasonography and mammography.

Level of evidence: intermediate; level of recommendation: recommended.

### Contraindications for breast MRI examinations

1. Pregnancy.
2. Ferromagnetic materials such as pacemakers and surgical metal clips in their bodies, and other materials, prohibit them from approaching a strong magnetic field.
3. Claustrophobia.
4. History of allergy to any MRI contrast agent, such as gadolinium chelate.
5. Very poor general condition, inability to lie prone, or intolerability to MRI examination.

### Technical specifications for breast MRI examination

#### Preparation before examination

##### Clinical history

4.3.1.2.1 Preparation before examination

Optimal examination time: Since the enhancement of normal breast tissue is most significant in the secretory phase of menstrual cycle, it is recommended that premenopausal women undergo MRI in the second week (days 7-14) of the menstrual cycle.

Level of evidence: intermediate; level of recommendation: recommended.

##### MRI examination

4.3.1.2.2 Equipment requirements

Scanners with high magnet field strength (1.5 T or above) can ensure better signal-to-noise ratio and fat suppression effectiveness in breast MRI examination. A dedicated breast coil must be used, and an open coil is recommended to allow lateral MRI-guided interventions if necessary.

4.3.1.2.3 Body position

The subject should lie prone, with both breasts hanging naturally in the center of the breast coil.

4.3.1.2.4 Imaging sequence

The scans generally include those of the transverse, sagittal, and coronal planes, with T1-weighed imaging (T1WI) nonfat suppression sequence, T2-weighed imaging (T2WI) fat suppression sequence, T1WI enhanced scan sequence diffusion-weighted imaging sequence. It is recommended that the *b* value be set to 800 s/mm^2^.

4.3.1.2.5 Postprocessing

Dynamic enhancement curves, maximum-intensity projection (MIP), and apparent diffusion coefficient (ADC) values should be recorded.

### Specifications of diagnostic report

The morphological features and the characteristics of dynamic enhancement curve of the lesion should be described. When describing the morphological features, signal characteristics on T1WI and T2WI before and after the enhancement should be comprehensively detailed. The morphology of the lesion should be described based on the morphology after the enhancement and categorized into three types: punctate enhancement, mass, and non-mass enhancement.

#### Punctate enhancement

Punctate enhancement refers to enhancement smaller than 5 mm, which can be benign. Fewer than 3% of the cases are likely malignant.

#### Mass

Mass refers to a space-occupying lesion with 3D space, with or without displacement or infiltration of surrounding normal tissue. Three aspects should be described: morphology, edge, and internal enhancement.

#### Non-mass enhancement

The classification of non-mass enhancement should be mainly based on three aspects: morphological characteristics, internal enhancement characteristics, and whether the lesion is bilaterally symmetrical.

#### Other signs and accompanying signs

Other signs include intramammary lymph nodes, skin lesions, and fat-containing lesions. Accompanying signs include such things as nipple retraction and involvement; skin thickening, retraction, and involvement; pectoral muscle involvement; and abnormal lymph nodes.

#### Lesion localization

1. First identify in which breast the lesion resides.
2. After positioning the lesion in the left or right breast, continue to position it in the following seven areas: the quadrants of upper lateral, lower lateral, upper medial, lower medial, posterior areola area, central area, and caudal lobe area.
3. Depth of the lesion.

### Composition of breast MRI report

It should contain a brief medical history of the patient, comparison with previous examinations, scanning specifications, fibroglandular composition of the breast, background parenchymal enhancement and relevant imaging findings, as well as evaluation classification and treatment recommendations. The wording of the report should be concise, using standard terms of the BI-RADS lexicon. The BI-RADS classification again contains seven categories (categories 0-6).

#### Incomplete assessment

BI-RADS 0: requires further imaging evaluation.

#### Assessment is complete

1. BI-RADS category 1: negative.
2. BI-RADS category 2: benign lesions.
3. BI-RADS category 3: probably benign; short-term follow-up examination recommended; very low likelihood of malignancy (< 2%).
4. BI-RADS category 4: suspicious malignant lesion. The likelihood of malignancy of such lesion is 2% to 95%. It can be subdivided into subcategories 4A, 4B, and 4C, whose malignancy probabilities are 2–10%, 10–50%, and 50–94%, respectively.
5. BI-RADS category 5: highly suggestive of malignancy (malignancy probability ≥ 95%). Clinical interventions should be adopted.
6. BI-RADS Category 6: known biopsy-proven malignancy that needs to be corrected with extended surgery. The purpose of MRI examination is to evaluate whether there is any residual lesion.

Level of evidence: high; level of recommendation: recommended.

## Guideline for image-guided breast histological biopsy

Specifically, it includes image-guided core-needle biopsy, vacuum-assisted biopsy, and wire-localized surgical biopsy.

### Indications

#### Ultrasound-guided breast lesion biopsy

1. Breast ultrasound finds suspicious space-occupying breast lesions that have not been palpated, of BI-RADS ≥ category 4 or some category 3 lesions. Biopsy can also be considered if necessary.

Level of evidence: high; level of recommendation: recommended.

(2) The breast mass can be palpable, and ultrasound indicates that there is a space-occupying lesion in the corresponding part of the breast, for which minimally invasive biopsy or minimally invasive resection is required to confirm the diagnosis.

Level of evidence: intermediate; level of recommendation: recommended.

#### Mammogram-guided breast lesion biopsy

1. The breast mass is not palpable, but mammography shows suspicious small, calcified lesions of BI-RADS ≥ category 4.
2. The breast mass is not palpable, but lesions of other types of BI-RADS ≥ category 4 (e.g., mass, architectural distortion, etc.) are found through mammography and cannot be accurately located through ultrasound.
3. For some category 3 lesions, if they are suspicious, then biopsy can also be considered.
4. The breast mass is palpable on physical examination, mammography shows that there is a space-occupying lesion in the corresponding position, and minimally invasive biopsy or minimally invasive resection is required to confirm the diagnosis.

Level of evidence: high; level of recommendation: recommended.

#### Other

If conditions permit, image-guided minimally invasive biopsy (core-needle biopsy or vacuum-assisted biopsy) should be actively adopted before surgery. Otherwise, direct image-guided wire-localized surgical biopsy can be considered.

Level of evidence: intermediate; level of recommendation: recommended.

### Requirements for image-guided breast biopsy equipment

#### Mammogram-guided breast biopsy

Mammography stereotactic bed or mammography machine equipped with a localized biopsy device.

#### Ultrasound-guided breast biopsy

High-frequency breast ultrasound probe: frequency: 7–15 Hz.

#### MRI-guided breast biopsy

For lesions found on MRI but not on mammogram or ultrasound, it is recommended to use ultrasound to review them first. If the lesion is still found in the corresponding position on ultrasound, it is recommended to perform an ultrasound-guided biopsy. If the ultrasound examination fails to find the lesion, an MRI-guided biopsy can be performed if conditions permit.

#### Guidewire-localized surgical biopsy

Single-hook or double-hook steel guidewire (recommended specification: 20–22 G).

#### Minimally invasive biopsy equipment

Core-needle-injection biopsy instrument (recommended specification: 14 G); vacuum-assisted breast biopsy system (recommended specification: 8-11 G).

Level of evidence: intermediate; level of recommendation: recommended.

### Image-guided guidewire-localized surgical biopsy

#### Contraindications

Severe systemic diseases or severe bleeding diseases.

#### Preoperative preparation

1. Have the patient sign the informed consent form.
2. Check and confirm the image data. It is recommended that clinicians use a marker to outline the approximate location of the lesion on the mammogram or breast. Surgical incisions can be marked for patients with breast-conserving surgery and skin-saving mastectomy.
3. Check the image positioning equipment to ensure the accuracy and precision.
4. Preoperative blood routine and coagulation function indicator tests.

#### Intraoperative precautions

1. During the operation, the localizing guidewire should be placed at the central part of the lesion under imaging guidance.
2. The locations of lesion and puncture needle under imaging guidance should be recorded through photograph or video and then keep it on file.
3. The tissue biopsy puncture needle path and localizing guidewire insertion point should be within the surgical incision marked by the surgeon as much as possible.
4. A circular breast tissue within a radius of at least 2 cm from the top of the localizing wire should be intraoperatively resected.
5. A biopsy specimen of a tiny calcification should be photographed immediately. After the operator confirms that the lesion has been biopsied, the specimen image should be sent for pathological examination together with the specimen.

Level of evidence: intermediate; level of recommendation: recommended.

### Image-guided minimally invasive breast biopsy

#### Contraindications

Those with severe systemic diseases or severe bleeding diseases.

#### Preoperative preparation

1. Signing of the informed consent form.
2. Check and confirm the imaging data, reposition the lesion on the mammogram and breast ultrasound images, and make corresponding markings.
3. Check all image guidance equipment and minimally invasive biopsy equipment to ensure accuracy and precision.
4. Preoperative blood test indicators: blood routine and coagulation function.

#### Intraoperative precautions

1. Select the incision, adopt the principle of proximity, and consider the aesthetics after biopsy.
2. The locations of the lesion and puncture needle under imaging guidance should be recorded through photographs or video, which should be kept on file.
3. Take a large enough specimen to ensure a pathological diagnosis. If conditions permit, a metal marker should be placed at the biopsy site.
4. Compress the surgical site for 5-15 minutes after biopsy.

#### Treatment of breasts and specimens after surgery

1. Compression bandaging should be kept on for at least 24 h after surgery. In case of ecchymosis or hematoma, the bandaging can be extended for 1 to 2 days. Generally, ecchymosis or hematoma will subside in 2 to 4 weeks.
2. When collecting biopsy specimens of microcalcification, mammography should be immediately done to confirm that the lesions have been biopsied.
3. Pack the specimen strips with calcification and those without calcification separately, and place them into different containers. Fix them with 4% formaldehyde solution, and send them for inspection [11].

Level of evidence: intermediate; level of recommendation: recommended.

## Part 4: Treatment: Early-stage breast cancer

### Clinical guidelines for breast-conserving therapy in invasive breast cancer

#### Indications for breast-conserving therapy

It is mainly aimed at patients who want to preserve the breast and have no contraindications for breast conservation.

#### Early-stage breast cancer (clinical stages I and II)

Patients with early-stage breast cancer who are at stages T_1_ and T_2_ in terms of tumor size, with appropriate breast size and appropriate tumor-to-breast volume ratio, and can still have a good breast shape after surgery. For those with multifocal breast cancer (i.e., multiple lesions in the same quadrant), breast-conserving surgery may also be attempted [12–14].

Level of evidence: high; level of recommendation: recommended.

#### Patients of clinical stage III breast cancer (excluding those with inflammatory breast cancer)

This can also be carefully considered when the patient meets the standards of breast-conserving surgery after preoperative treatment and downstaging.

Level of evidence: high; level of recommendation: recommended [12, 15, 16].

#### Absolute contraindications for breast-conserving therapy

1. During pregnancy, breast-conserving surgery can be done, while radiotherapy must wait until after delivery.
2. Lesions so extensive that it is difficult to achieve negative margins or ideal breast-conserving appearance.
3. Diffuse distribution of malignant calcification foci.
4. Tumors that have a positive margin after extensive local resection and no guaranteed negative pathological margin after reresection.
5. Refusal of breast-conserving surgery.
6. Inflammatory breast cancer.

#### Breast-conserving surgery should be considered cautiously when the following factors are involved:

1. Active connective tissue disease, especially scleroderma, systemic lupus erythematosus, or collagen vascular disease, who have poor tolerance to radiotherapy.
2. For patients who have previously received breast or chest wall radiotherapy on the ipsilateral breast, the dose and range of the radiotherapy should be known.
3. Those with a tumor > 5 cm in diameter or a high tumor-to-breast volume ratio are prone to have conflict between satisfactory appearance and adequate margins.
4. Multicentric lesions (multicentric lesions refer to the presence of one or more lesions in two or more quadrants, or two breast lesions with completely different pathological types and molecular types).
5. Involvement of the nipple (e.g., Paget’s disease of the nipple).
6. When the resection margin is close, and the distance between the ink-stained incision margin and the tumor is shorter than 2 mm (invasive cancer, except for those who cannot be re-resected on the surface and base). There is no consensus on the specific criteria of a "close resection margin", and most experts tend to agree that when the distance between the resection margin and the tumor is 2 mm, it may affect the local control of breast-conserving therapy patients.
7. A strong genetic susceptibility to breast cancer (e.g., *BRCA1*/*2* gene mutation) and an increased risk of recurrence in the ipsilateral breast after breast preservation.

### Postoperative pathological examination

1.2.1 Gross examination of the resection margin of the lesion and measurement of the distance between the resection margin and the tumor under microscope. It is recommended to simultaneously report the direction and distance of the nearest resection margin as well as the tumor type.

1.2.2 In other aspects, it is the same as routine pathological examination.

1.2.3 When the postoperative pathology report suggests that there is pleomorphic lobular carcinoma in situ or ductal carcinoma in situ on the resection margin, further extensive resection is recommended to ensure that the resection margin is negative. It is not recommended to replace extensive resection with local radiotherapy [17].

1.3 Indications for radiotherapy after breast-conserving surgery for breast cancer

In principle, all patients undergoing breast-conserving surgery require radiotherapy.

Level of evidence: high; level of recommendation: recommended.

However, for patients who simultaneously meet the following conditions, after weighing the absolute and relative benefits of radiotherapy and fully considering the convenience to the patient, systemic concomitant diseases, and the patient’s wishes, exemption from radiotherapy can be considered:

1. The patient is ≥ 70 years old.
2. The tumor has a pathological stage of T_1,_ N_0_ , or M_0_.
3. The tumor is hormone receptor positive.
4. The patient has negative resection margins and can receive standard endocrine therapy.

Level of evidence: intermediate; level of recommendation: recommended.

## Clinical guidelines for sentinel lymph node biopsy (SLNB) for breast cancer

### Indications for SLNB

SLNB is a standard axillary staging method for early invasive breast cancer. See Table 1 for specific indications. It is currently believed that the contraindications for SLNB in patients with operable breast cancer include only patients with inflammatory breast cancer and those who have a confirmed metastasis through axillary lymph node puncture and have not received neoadjuvant therapy or have a positive axillary lymph node and remain positive after neoadjuvant therapy. The accuracy and safety of SLNB in ​​patients with clinically negative axillary lymph nodes after the cN_2-3_ neoadjuvant treatment remain to be verified. Patients with positive or negative axillary lymph nodes can proceed to intramammary SLNB [18, 19].

**Table 1:** Indications for SLNB

Indication	Controversial indication	Contraindication
Early invasive breast cancer		Inflammatory breast cancer
Clinical axillary lymph node negative ^a^	Intraductal cancer with breast-conserving surgery ^e^	Positive axillary lymph node in clinical examination, confirmed by puncture biopsy
	cT_1_ N_0_, age > 70, luminal A, with concomitant diseases ^f^	Positive axillary lymph node and remains positive after neoadjuvant therapy
Unifocal or multifocal disease		Clinically negative axillary lymph node after cN_2-3_ neoadjuvant therapy
No sex limitation		
No age limitation	Patients with ipsilateral recurrence/relapse after breast-conserving surgery ^g^	
Intraductal cancer with mastectomy ^b^		
Negative axillary lymph node after neoadjuvant therapy		
Puncture-confirmed clinical axillary lymph node-negative after cN_1_ neoadjuvant therapy ^c^		
Pregnancy ^d^		

^a^Suspicious axillary lymph nodes on clinical examination and imaging examination can be assessed by ultrasound-guided fine-needle aspiration or core-needle biopsy. Patients with negative cytology or histopathology results can still proceed to the SLNB process. ^b^ If there is no upgrade from intraductal cancer to invasive cancer as confirmed by resection biopsy, SLNB can be waived. ^c^ Must be in line with sentinel lymph nodes that align with the placement label of puncture-confirmed positive lymph nodes before neoadjuvant therapy, through the dual tracer method, and these include the labeled lymph nodes. ^d^ SLNB with radionuclide tracer is safe to fetuses, but blue dye tracer is not recommended due to possible allergies. ^e^ If the resection of the primary breast tumor does not affect the success rate or accuracy of subsequent SLND, the same-stage SLNB may be skipped. ^f^ If SLNB is not performed, the sentinel biopsy can be waived, and no axillary treatment will be performed. ^g^ The accuracy and safety of the second SLNB for patients with ipsilateral breast recurrence/relapse after breast-conserving surgery combined with SLNB have been preliminarily confirmed.

### Intraoperative confirmation and detection of SLNs

Whether mastectomy or breast-conserving surgery will be done, SLNB should generally precede breast surgery, especially when the blue-dye tracer alone is used. Depending on the tracer, the intraoperative determination method of SLNs varies. The dye method requires the detection of the first blue-stained lymph node out of all blue-stained lymphatic vessels. Detection of all blue-stained lymphatic vessels is essential to avoid missed SLNs and lower the false-negative rate. The threshold for SLNs under the radionuclide method is all lymph nodes that exceed 10% of the highest lymph node count. During the operation, the probe of the gamma detector should be moved slowly, in an orderly manner, and the lymph nodes should be accurately counted by placing the probe close to lymph node. After the SLN is detected using the dye method and/or nuclide method, the axillary region should be palpated, and the enlarged hard lymph nodes found through palpation should also be separately sent for SLN examination.

### Criteria for determining the type of SLN metastasis, prognostic significance, and clinical treatment

#### Prognostic significance and axillary treatment of different types of SLN metastasis

1. Macrometastases: Approximately 50% of patients are positive on axillary nonsentinel lymph nodes (nSLNs). Axillary lymph node dissection (ALND) is one of the standard treatments, and the further prognostic data obtained through ALND can change the treatment decision. Breast-conserving therapy patients of clinical stage T_1-2_ who have not received neoadjuvant therapy, have negative axillary lymph nodes, but have 1-2 SLN macrometastases on pathological examination and will receive subsequent further adjuvant whole-breast radiotherapy and systemic therapy can be exempt from ALND. For patients with 1-2 SLN macrometastases who have undergone mastectomy, axillary radiotherapy may be a reasonable alternative to ALND if the prognostic data obtained from ALND do not change the treatment decision and the patient agrees to no ALND.

Level of evidence: high; level of recommendation: recommended.

(2) Micrometastases: 13% to 20% of patients are positive in axillary nSLNs, and approximately 10% have macrometastases. ALND can lead to upstaging in 15% of patients, and the adjuvant therapy changes in 7%. When receiving breast-conserving therapy (combined with whole-breast radiotherapy), patients with SLN micrometastases can be exempted from ALND. For patients with SLN micrometastases who undergo total mastectomy without radiotherapy, the opinions of most Chinese experts are in favor of an axillary treatment identical to that for patients with macrometastases.

Level of evidence: high; level of recommendation: recommended.

(3) Isolated tumor cells (ITCs): The probability of axillary nSLN metastasis is lower than 8% (invasive ductal carcinoma larger than 5 mm), and ALND can lead to upstaging in 4% of the patients. At present, it is believed that ITCs have an adverse effect on prognosis, and the patients can benefit from systemic adjuvant therapy as patients with micrometastases do. However, the axillary recurrence rate of ITC patients who do not receive axillary therapy does not significantly increase, so routine ALND is not recommended.

Level of evidence: high; level of recommendation: recommended.

(4) Initial SLN negativity: no need for axillary treatment.

Level of evidence: high; level of recommendation: recommended.

(5) Neoadjuvant therapy:
  ① Patients with negative initial clinical axillary lymph nodes: SLN-negative patients can avoid ALND. ALND is still the standard treatment for SLN-positive patients, including those with macrometastasis, micrometastasis, or ITCs. Axillary radiotherapy can be considered as an alternative to ALND for patients with one SLN macrometastasis, micrometastasis, or ITC before neoadjuvant treatment. It is recommended that SLNB after neoadjuvant therapy be the first choice. For patients who have undergone SLNB before neoadjuvant therapy and have been pathologically confirmed as SLN negative, if the clinical lymph node is negative after neoadjuvant therapy, no surgical evaluation of axillary status is needed. ALND can be skipped in patients who have undergone SLNB before neoadjuvant therapy and have been pathologically confirmed to have clinical stage T_1-2_ breast cancer with 1-2 positive SLNs, those who have shown an effective response to neoadjuvant therapy and plan to receive whole-breast radiotherapy after breast-conserving surgery, and those who receive posterior axillary radiotherapy after mastectomy. ALND is the standard axillary management for patients with three or more positive SLNs detected by SLNB before neoadjuvant therapy. It is not recommended to perform two SLNBs (such as before and after neoadjuvant therapy).
  ② Not all patients with positive clinical lymph nodes are suited for neoadjuvant therapy before SLNB after downstaging. The effectiveness of lymph node biopsy after neoadjuvant therapy in patients with clinical lymph node stage of cN_2_ or above still lacks evidence from large samples. For patients at stage cN_1_ before neoadjuvant therapy, preserving the axilla by downstaging through neoadjuvant therapy is more suitable. SLN-negative patients who meet the following conditions can avoid ALND after the options have been communication with them: stage cT_1-3_N_1_, detection by two tracers on imaging, detection of ≥ 3 SLNs, and a puncture biopsy–confirmed positive axillary lymph node has been placed with a marking clip before neoadjuvant chemotherapy and is detected intraoperatively. For patients who underwent SLNB after neoadjuvant therapy and were confirmed as negative (YPN_0_), most experts recommend adjuvant radiotherapy in the range of axillary groups I and II after surgery. For patients with puncture-confirmed stage cN_1_ tumors that are histopathologically confirmed as SLN metastases (including macrometastases, micrometastases, and ITCs) after neoadjuvant therapy, ALND should be performed. For patients with puncture-proven cN1 tumors for which neoadjuvant therapy has failed, ALND is still the optimal axillary management.

Level of evidence: intermediate; level of recommendation: recommended.

### Follow-up of patients with ALND-replacing SLNB

In addition to the routine review items, ultrasound examinations of bilateral axillary and clavicle areas should be routinely performed, and MRI examination can be considered if conditions permit. If abnormal axillary lymph nodes are found in clinical or ultrasound examination, ultrasound-guided fine-needle aspiration or core-needle biopsy should be performed, and open biopsy should be performed if necessary [18, 19].

## Clinical guidelines for breast reconstruction and plastic surgery

### Purpose of breast reconstruction

Breast reconstruction can help patients reconstruct the shape, contour, and anatomical landmarks of the breast and maintain their body shape while maximally achieving basic symmetry of the breast shapes on both sides.

### Indications for breast reconstruction

Breast reconstruction is suitable for women who are preparing for or have received mastectomy for various reasons, or those with breasts significantly deformed due to breast-conserving surgery.

### Types of breast reconstruction

According to the timing of reconstruction, breast reconstruction can be divided into three categories: immediate reconstruction, delayed reconstruction, and staged immediate breast reconstruction.

According to the reconstruction material, breast reconstruction can be divided into three categories: autologous tissue (skin flap) reconstruction, implant reconstruction, and reconstruction with two combined materials (e.g., latissimus dorsi myocutaneous flap combined with implant).

### Relationship between postoperative radiotherapy and breast reconstruction

For patients who clearly need postoperative adjuvant radiotherapy, it is recommended to consider delayed or staged breast reconstruction. Radiotherapy may adversely affect the shape of the reconstructed breast and may even cause reconstruction failure. An experienced team can provide radiotherapy after immediate reconstruction after full communication with the patient. For this, it is generally recommended to use autologous tissue to reduce the impact of radiotherapy on breast reconstruction. When considering tissue expansion and immediate implant reconstruction, it is recommended to place the tissue expander first, and replace it with a permanent prosthesis before or after radiotherapy. Completing the prosthesis replacement surgery before radiotherapy can reduce incision-related complications and the risk of expander rupture during radiotherapy. If the tissue expander is replaced with a permanent prosthesis after the radiotherapy, it is recommended that the replacement surgery be performed 6 months after the radiotherapy, when the skin reaction caused by radiotherapy is relieved, as this strategy may improve the final aesthetic effect of the reconstructed breast. Breast reconstruction with anterior pectoral implants is more tolerant to radiotherapy, though this needs to be confirmed by larger studies. For patients who adopt the implant reconstruction after radiotherapy, severe cystic contracture, displacement, poor appearance of the reconstructed breast, and implant exposure can often happen. Therefore, it is not appropriate to use tissue expanders and implants for delayed breast reconstruction after radiotherapy. Instead, autologous tissue should be the first choice.

### Evaluation system after breast reconstruction

For the evaluation of the effect of breast reconstruction surgery, it is recommended to use the assessment tools that contain patient-reported outcomes. Before using foreign postoperative satisfaction evaluation scale for breast reconstruction, an authorized, Chinese, and reliability/validity-tested scale should be used for clinical research and clinical practice. A baseline survey of the patient is recommended preoperatively, followed by surveys 3 and 12 months after surgery and annually thereafter.

## Guidelines for the treatment of breast carcinoma in situ

### Treatment of lobular carcinoma in situ (LCIS) at initial diagnosis

#### Surgical treatment

The need for excisional biopsy when atypical lobular hyperplasia (ALH) or atypical LCIS is detected by core-needle biopsy is the consensus of most current research findings, with the main purpose of minimizing the coexistence risk of ductal carcinoma in situ (DCIS) and invasive cancer.

Pleomorphic LCIS may have similar biological behaviors as DCIS. Clinicians can consider complete resection of the lesion and negative margins, but this may lead to a high rate of total mastectomy without clinical benefit. The coexistence of LCIS and IDC or DCIS is not a contraindication for breast preservation.

#### Nonsurgical treatment

If no other cancerous changes are present, follow-up review can be considered for patients with LCIS after tumor resection. Radiotherapy is not recommended, and there are no data to support radiotherapy for pleomorphic LCIS.

#### Prophylactic treatment

##### Drug prophylactic treatment

Tamoxifen (20 mg/d, orally for 5 years) is considered an option for reducing the risk of invasive, estrogen receptor (ER)–positive breast cancer in premenopausal women. Administration of tamoxifen based on the patient’s ER status is currently an effective option for the prevention of ER-positive breast cancer. Tamoxifen (5 mg/d, orally for 3 years) is also optional for patients with a predicted low risk. Raloxifene (60 mg/d, orally for 5 years) can be an option to reduce the risk of invasive, ER-positive breast cancer, and its administration should be combined with ER testing, but it is only suitable for postmenopausal women. Exemestane (25 mg/d, orally for 5 years) and anastrozole (1 mg/d, orally for 5 years) are considered options for reducing the risk of invasive, ER-positive breast cancer in postmenopausal women.

For women over 35 years of age who are at high risk of breast cancer (including atypical lobular hyperplasia, atypical ductal dysplasia, LCIS, and DCIS confirmed by previous surgery), the possible use of the above four drugs can be considered, and the discussion can be based on risk factors such as age, family history, drug history, reproductive history, etc.

##### Preventive double mastectomy

As a high-risk factor for breast cancer, LCIS combined with other risk factors (e.g., family history, related *BRCA* gene mutations, etc.) can spur preventive double mastectomy. Currently, such surgery must be approved by an ethics committee.

### Treatment of DCIS at initial diagnosis

#### Local treatment

##### Surgery

According to the actual situation in China, if the resection margin is not assessed using the “ink staining" method, it is recommended that a negative margin first be ensured. If possible, a 2-mm negative margin should be ensured. In the case that parts of the base or surface margin are less than 2 mm and it is impossible to further increase the margin, a negative margin of less than 2 mm is also acceptable [14].

Level of evidence: high; level of recommendation: recommended.

##### Radiotherapy

For patients who are assessed by clinicians as having a low risk of recurrence, breast-conserving surgery can be performed without radiotherapy. For example, patients with low-grade DCIS who can be assigned to the low-risk group based on the van Nuys prognostic index (VNPI) can be exempted from adjuvant radiotherapy. However, this view is only supported by retrospective studies, while long-term follow-up results of the studies show that grouping by risk may only select patients with delayed recurrence times rather than those with low recurrence risk. Even for some patients with intermediate or low risk, the local recurrence rate after radiotherapy is significantly lower than that of patients without radiotherapy.

#### Systemic therapy: endocrine therapy

In the following situations, the use of tamoxifen for 5 years can be considered to reduce the risk of recurrence of ipsilateral breast cancer after breast-conserving surgery:

1. Patients who underwent breast-conserving surgery (lumpectomy) and radiotherapy, especially ER-positive DCIS patients; the efficacy of tamoxifen on ER-negative DCIS patients is still uncertain.
2. Patients who underwent only breast-conserving surgery. For DCIS patients with mastectomy, oral tamoxifen or raloxifene can reduce the risk of contralateral breast cancer, but the clinical benefits and adverse effects of chemoprevention need to be weighed.

Postmenopausal DCIS patients after surgery (including breast-conserving surgery and total mastectomy) may consider aromatase inhibitors (AIs) to prevent and reduce the risk of contralateral breast cancer.

### Reference for the selection of the breast DCIS treatment method

Some foreign scholars use VNPI as an objective indicator to help clinicians make decisions about the treatment of DCIS. VNPI comprehensively considers four aspects of DCIS: tumor size, patient age, surgical margin, and tumor cell nuclear grade. On each aspect, a score (1= best; 3 = worst) is obtained, from which the total score (4= best; 12 = worst) is obtained. Patients with a VNPI score of 10-12 are recommended to undergo total mastectomy, those with a VNPI score of 4-6 are recommended to undergo simple local excision, and those with a VNPI score of 7-9 are recommended to undergo extensive local excision combined with whole-breast radiotherapy. The specific scoring method of VNPI is detailed in Table 9 in Appendix.

Note: At present, the clinical application value of VNPI is still controversial, so it is only for clinicians' reference.

## Guidelines for systemic treatment of early-stage breast cancer

### Clinical guidelines for systemic adjuvant treatment after breast cancer surgery

#### Choice of systemic adjuvant therapy after breast cancer surgery

The choice of systemic adjuvant treatment after breast cancer surgery should be based on the individualized assessment of the risk of recurrence, the molecular classification of tumor pathology, and the patient’s expected response to different treatment options.

The classification of the risk of recurrence after breast cancer surgery is shown in Table 2. This table can be used to comprehensively assess the recurrence risk of patients after surgery and is an important basis for formulating systemic adjuvant treatment programs. The molecular classification of breast cancer is shown in Table 3. Physicians should choose an appropriate chemotherapy, endocrine therapy, and anti-human epidermal growth factor receptor 2 (HER2) therapy based on the patient's molecular classification and risk of recurrence [10, 14, 20–31].

**Table 2:** Classification of risk of recurrence after breast cancer surgery

Risk	Criterion		
	Metastatic lymph nodes	ER/PR	Other conditions
Low risk	Negative	Positive	Simultaneously meet the following conditions ^a^: pT ≤ 2 cm; histological grade I; LVI negative; HER2 negative; age ≥ 35 years; Ki67 ≤ 20% or laboratory median value
			Negative HER2; do not meet the above conditions, but polygenic testing result indicates low risk
Intermediate risk	Other situations that do not meet the definition of low or high risk		
High risk	1–3 positive	Positive	Meet any of the following conditions ^b^: histological grade III; pT > 5 cm; HER2 positive; polygenic testing result high-risk
		Negative	Any condition
	≥ 4 positive	Any condition	Any condition

^a^Polygenic testing is unnecessary at this time (e.g., 21-gene or 70-gene).

^b^Although Ki67 is one of the independent factors for breast cancer recurrence, the expert panel is still undecided whether pN1 with high Ki67 expressed is conclusively high-risk. Although pN_1_ with high Ki67 is the classification criterion of high-risk luminal-HER2 negative breast cancer in certain clinical trials, this classification does not have universality.

**Table 3:** Marker detection and determination of molecular typing of breast cancer

Intrinsic molecular typing	IHC4-based molecular typing	Note
Luminal Type A	Luminal A-like ER/PR positive and PR high expression/HER2 negative Low Ki-67 proliferation index	It is recommended that the criteria of ER/PR expression, and Ki-67 proliferation index be reported in terms of the percentage of positive cells. The criterion of the Ki-67 proliferation index may vary in different pathological experimental centers, and 20–30% can be used as the threshold for determining high or low Ki-67, while, 20% can be the threshold for determining the level of PR expression ^a^ to further distinguish between luminal A and luminal B (HER2 negative).
Luminal Type B	Luminal B-like (HER2 negative) ER/PR positive HER2 negative and high Ki-67 proliferation index or low PR expression	The abovementioned luminal-like tumors that do not meet the conditions of the luminal A-like type can be regarded as the luminal B-like subtype
	Luminal B-like (HER2 positive) ER/PR positive HER2 positive (protein overexpression or gene amplification) Ki-67 in any state	
ERBB2 + type	HER2 positive HER2 positive (protein overexpression or gene amplification) ER negative and PR negative	
Basal-like type	Triple-negative (nonspecial invasive ductal carcinoma) ER negative PR negative-HER2 negative	The overlap between triple-negative breast cancer and basal-like breast cancer is approximately 80%, but triple-negative breast cancer also includes some special types of breast cancer such as medullary carcinoma (typical) and adenoid cystic carcinoma.

^a^20% is used as the threshold for determining the level of progesterone receptor (PR) expression, which is currently supported by only one retrospective study (J Clin Oncol, 2013, 31: 203-209)

### Clinical guidelines for adjuvant chemotherapy after breast cancer surgery

#### Population selection for adjuvant chemotherapy after breast cancer surgery

See Table 4 for population selection guidelines of adjuvant chemotherapy after surgery.

**Table 4:** Patients to whom postoperative adjuvant chemotherapy after surgery is recommend

Risk of recurrence	Luminal HER2 negative	HER2 positive	Triple negative
Low risk	Waiver of chemotherapy	n/a	n/a
Intermediate risk and pN_0_^b^	• Chemotherapy is recommended for T _3_ or above; • T_1b-2_: Subjected to 21-gene or 70-gene testing ① 21-gene testing: if age > 50 and RS > 25, then chemotherapy is recommended ② 21-gene testing: if age ≤ 50 and RS ≥ 16, then chemotherapy is recommended ③ 70-gene testing: if clinical data ^a^ and 70-gene test both show high risk, then chemotherapy is recommend ④ 70-gene testing: if clinical risk is high ^a^ and 70-gene test shows low risk and age ≤ 50, then chemotherapy should be considered •T_1b-2_: Those who have not done genetic testing and have any of the following characteristics can be considered for chemotherapy: low ER expression, histology grade 3, LVI positive, age < 35, high Ki67 • T_1a_: Chemotherapy can be generally waived, and when multiple risk factors are present, it should be considered comprehensively in an individualized manner.	• Chemotherapy is recommended if T_1b_ or above • Chemotherapy should be considered if T_1a_^d^ • Chemotherapy is generally not considered if T_1mic_, requiring further individualized comprehensive consideration	• Chemotherapy is recommended if T_1b_ or above • Chemotherapy should be considered if T_1a_ • Chemotherapy is generally not considered if T_1mic_
Intermediate risk and pN_1_	• Chemotherapy is recommended • Unless T_1-2_ and having the following 21-gene or 70-gene test results, chemotherapy waiver should be considered: ① 21-gene test: patients with RS ≤ 11 ^e^; ② 70-gene test: clinical high risk ^a^, 70-gene low-risk, and age > 50	n/a	n/a
High risk	Any	Any	Any

^a^Based on the evaluation of the simplified version of Adjuvant! Online

^b^ITCs of pathological lymph nodes are managed the same way as pN_0_, and pN_1 mic_ is managed the same way as pN_1_

^c^The expert group believes that it is not yet possible to determine whether chemotherapy is needed based on the Ki67 index alone, but the higher the Ki67 index, the stronger the indication for chemotherapy.

^d^Anti-HER2 monoclonal antibody therapy can be considered for T_1a_, but there is no consensus on whether to combine it with intravenous chemotherapy. See the molecular subtype section for specific regimens.

^e^At present, this is mainly based on the data of the PLAN-B trial. Although the RXPONDER trial confirmed that postmenopausal patients with pN_1_ and recurrence score (RS) < 25 may be exempt from chemotherapy, the results of that study need to be further verified and recognized.

Multigene panel testing tools (e.g., Oncotype DX ®, MammaPrint ®, etc.) can help guide adjuvant chemotherapy decisions, but testing tools with the corresponding qualifications are recommended. For patients who are unqualified or unwilling to receive multigene panel testing, whether to adopt adjuvant chemotherapy should be decided by comprehensively considering the clinicopathological characteristics of the tumor, the patient's physiological conditions and underlying diseases, and the possible benefits of and adverse reactions to chemotherapy [32, 33].

##### Contraindications of adjuvant chemotherapy after breast cancer surgery

1. Pregnancy: Chemotherapy is usually contraindicated for patients in the first trimester and should be selected carefully for patients in the second trimester.
2. Patients with obvious fatigue or cachexia.
3. Patients with severe infection, hyperthermia, or water-electrolyte or acid–base balance disorders.

(5) Patients with gastrointestinal obstruction or perforation.
(6) Patients with low bone marrow reserve; leukocytes ≤3.5×10^9/^L, or platelets ≤ 75×10^9/^L before treatment.
(7) Patients with serious damage to cardiovascular, liver, or kidney function.

##### Regimens of adjuvant chemotherapy after breast cancer surgery (Table 10 in Appendix)

Commonly used regimens are as follows: ① Regimens based on anthracyclines, such as adriamycin (doxorubicin)/cyclophosphamide (AC) and epirubicin/cyclophosphamide (EC). Although the evidence-based medical data on pirarubicin (THP) are limited, it is feasible to replace doxorubicin with THP in daily clinical practice in China, at a recommended dose of 40-50 mg/m^2^. The value of 5-fluorouracil in adjuvant therapy has gradually been deprecated. ② Combination of anthracyclines and taxanes, such as AC + docetaxel. ③ Sequential regimens of anthracycline and taxanes, such as AC→paclitaxel (once a week), AC→docetaxel (once every 3 weeks), dose-intensive AC followed by paclitaxel (once every 2 weeks), and dose-intensive AC followed by paclitaxel (once a week). According to the CALGB 9741 study and the EBCTCG meta-analysis, dose-intensive chemotherapy can bring more benefits to patients. Therefore, for patients with triple-negative breast cancer and positive lymph nodes, dose-intensive chemotherapy is preferred. ④ Combination chemotherapy regimens without anthracyclines: the TC regimen (four or six courses of docetaxel/cyclophosphamide) is suitable for patients with a certain risk of recurrence, contraindications, or intolerance to anthracyclines; the PC regimen (weekly paclitaxel/carboplatin; see the PATTERN study) can be considered for patients with triple-negative breast cancer; the CMF regimen (cyclophosphamide/methotrexate/5-fluorouracil) is rarely used. ⑤ Intensive capecitabine (combined or sequential) can be considered for triple-negative breast cancer. For example, in the CBCSG010 study, the sequence of anthracycline and docetaxel was used in combination with capecitabine. In the SYSUCC001 study, capecitabine was used alone for one year after adjuvant intravenous chemotherapy. In the CREATE-X study, eight courses of capecitabine alone were used on the non-pathologic complete remission (non-pCR) population after neoadjuvant chemotherapy. ⑥ Intensive olaparib in patients with a high risk of pathogenic/suspected pathogenic g*BRCA* mutations. The OlympiA study suggested that HER2-negative patients who have non-pCR after neoadjuvant therapy or directly operated-on triple-negative breast cancer [≥ pT _2_ and (or) ≥ pN_1_] and luminal type (≥ pN_2_) had significantly improved 3-year invasive disease-free survival after 1 year of olaparib, but this drug has not yet been indicated for adjuvant therapy. ⑦ Albumin-bound paclitaxel can be tried to replace paclitaxel or docetaxel when it is medically necessary (e.g., reducing allergic reactions, etc.), but its weekly dose should not exceed 125 mg/m^2^ [32, 34–44]

Level of evidence: high; level of recommendation: recommended.

(2) For common regimens for HER2-positive breast cancer, see section 5.1.4: clinical guidelines for adjuvant anti-HER2 therapy after breast cancer surgery.

#### Clinical guidelines for adjuvant endocrine therapy after breast cancer surgery

##### Population selection for adjuvant endocrine therapy after breast cancer surgery

All ER- and/or PR-positive breast cancer patients should receive postoperative adjuvant endocrine therapy. According to the latest guidelines of American Society of Clinical Oncology/College of American Pathologists, although tumors with 1% to 100% ER immunohistochemistry (IHC) staining are considered ER positive, 1–10% of ER IHC staining is of low ER expression. The biological behavior of low-ER-expressing breast cancer is usually similar to that of ER-negative breast cancer, and this should also be taken into consideration when making treatment decisions [27, 45].

Level of evidence: high; level of recommendation: recommended.

##### Regimens of adjuvant endocrine therapy after breast cancer surgery

Regimens of adjuvant endocrine therapy for premenopausal patients:

1. There are three options for adjuvant endocrine therapy: tamoxifen, ovarian function suppression plus tamoxifen, and ovarian function suppression plus third-generation AI. Ovarian function inhibitors are recommended for patients with a high risk of recurrence, while the patient’s age, tumor size, lymph node status, histological grade, etc., should be considered, and the Screening Tool for Early Predictors of Posttraumatic Stress Disorder (STEPP) score evaluation can also be used. For young (< 35 years old) breast cancer patients, ovarian function suppression plus AI is preferred [46, 47].

Level of evidence: high; level of recommendation: recommended.

(2) For patients taking tamoxifen, after 5 years of tamoxifen use, some patients (e.g., those with a high risk of relapse) may consider extending the use to 10 years if still premenopausal. There is no evidence that premenopausal patients who have taken tamoxifen for 5 years will further benefit from ovarian function inhibitors combined with a third-generation AI [46, 47].

Level of evidence: high; level of recommendation: recommended.

(3) The methods of ovarian suppression include drug-induced castration, surgical removal of the ovaries, and ovarian radiation (drug-induced ovarian castration is recommended as the first choice). If drug-induced ovarian function suppression is used, the current recommended treatment duration is 5 years, but intermediate-risk patients can also choose to use it for 2 to 3 years. For extremely high-risk premenopausal patients who have been treated with drug-based ovarian function inhibitors + tamoxifen/AI for 5 years, although there is no strong evidence, follow-up tamoxifen monotherapy can also be considered, or they can continue with the original regimen, i.e., an extended original regimen [46, 47].

Level of evidence: intermediate; level of recommendation: recommended.

(4) After 5 years of tamoxifen use, high-risk patients can continue to take AI for 5 years while postmenopausal and can continue to take tamoxifen for 10 years if they are not in menopause [46, 47].

Level of evidence: high; level of recommendation: recommended.

(5) AIs and luteinizing hormone-releasing hormone analogues can lead to decreased bone mineral density (BMD) or osteoporosis, so BMD examination is routinely recommended before and during the use of these drugs. BMD is measured once every 12 months, at which times the BMD score (T-score) should be taken [46].

Level of evidence: intermediate; level of recommendation: recommended.

Adjuvant endocrine therapy for postmenopausal patients:

1. Third-generation AIs can be recommended to all postmenopausal ER- and/or PR-positive patients, especially the following: ① patients at high risk of recurrence; ② patients with contraindications to tamoxifen or patients with moderate or severe adverse reactions to tamoxifen; ③ high-risk patients who have taken tamoxifen 20 mg/d × 5 years [48].

Level of evidence: high; level of recommendation: recommended.

(2) AIs (letrozole, anastrozole, exemestane) can be used for 5 years from the very beginning. Five years of adjuvant endocrine therapy is usually recommended for stage I patients. For patients with stage II lymph node-negative breast cancer, if the 5-year tamoxifen regimen 5 years is selected at the very beginning, 5 years of AI or tamoxifen is also recommended; if the patient is initially treated with a regimen of AI for 5 years, or treated with tamoxifen for 2 to 3 years and then switches to AI for the rest of the 5 years, extended endocrine therapy is not routinely recommended. For patients with stage II lymph node–positive breast cancer, regardless of their endocrine therapy regimen in the first 5 years, extended therapy with an AI for the next 5 years is recommended. For stage III patients, 5 years of extended therapy with an AI is recommended. For patients on extended therapy, the endocrine therapy can last 8 to 10 years [48].

Level of evidence: high; level of recommendation: recommended.

(3) For patients with ER^+^ breast cancer with ≥ 4 positive lymph nodes, regardless of menopausal status, adding 2 years of an intensive regimen of the CDK4/6 inhibitor abemaciclib to the standard adjuvant endocrine therapy can be considered; for ER^+^ patients with G3/T3/Ki67 ≥ 20%, at least one high-risk factor, and 1–3 positive lymph nodes, the intensive abemaciclib regimen can also be considered [48].

Level of evidence: intermediate; level of recommendation: recommended.

#### Clinical guidelines for adjuvant anti-HER2 therapy after breast cancer surgery

##### Population selection for adjuvant anti-HER2 therapy after breast cancer surgery

Refer to Table 5 for adjuvant treatment strategies for HER2-positive patients.

1. HER2 positivity refers to an IHC test result of 3+, or IHC 1+ and/or 2+ and a positive in situ hybridization (ISH) test result [29, 49].

**Table 5:** Adjuvant therapy strategies for HER2-positive patients

Lymph node status	Lump size	Regimen
Lymph node negative	T_1b_ or above (can be considered if T_1a_)	Adjuvant chemotherapy + trastuzumab
Lymph node micrometastasis (lymph node metastasis ≤ 2 mm)	Any	
Lymph node positive (≥ 1 ipsilateral metastasis; metastasis > 2 mm)	Any	Adjuvant chemotherapy + trastuzumab + pertuzumab; adjuvant chemotherapy + trastuzumab if conditions do not permit

For the interpretation of ISH test results, refer to Table 6.

**Table 6:** Interpretation of ISH test results

HER2/CEP17 ratio	Average HER2 copy number	ISH status interpretation
< 2.0	< 4.0	Negative
	≥ 4.0 and < 6.0	It is recommended to recount 20 cells, and if the result changes, then comprehensively analyze the two results; if the result remains unchanged and the IHC is 2+, it is deemed negative.
	≥ 6.0	It is recommended to increase the number of counted cells; if the result remains unchanged, it is deemed positive
≥ 2.0	< 4.0	It is recommended to increase the number of counted cells; if the result remains unchanged, it is deemed negative
	≥ 4.0	Positive

Low HER2 expression means IHC 1+ and/or 2+ and negative ISH.

##### Adjuvant anti-HER2 treatment regimens after breast cancer surgery

1. Application of trastuzumab in the adjuvant treatment of HER2-positive patients: in the case of negative lymph nodes, primary infiltrates > 0.5 cm and ≤ 2 cm, and HER2 positivity, trastuzumab is recommended, and weekly paclitaxel or TC×4 + trastuzumab adjuvant therapy (here C refers to CTX) can be considered. In the case of negative lymph nodes and primary tumor < 0.5 cm, trastuzumab can be considered, although it is only supported by limited evidence. For patients with a small tumor but lymph node micrometastasis, weekly paclitaxel or TC×4 + trastuzumab adjuvant therapy can be considered. When determining whether to choose short-course chemotherapy combined with trastuzumab for small HER2-positive tumors, individualized treatment regimens should be considered, in which the size of the invasion, ER status, and patient age are all factors going into the decision. The recommended trastuzumab treatment duration is still 1 year, which can be combined with chemotherapy or given after chemotherapy. Combined use with chemotherapy is preferred. Only in the PERSEPHONE study has the 6-month short-course been confirmed to be noninferior to the 1-year course, and the 2-year course of treatment does not achieve more prognostic benefit, so the two long courses (i.e., 1-year and 2-year courses) are not recommended at this time [50–55].

Level of evidence: high; level of recommendation: recommended.

(2) For patients with a high risk of recurrence (e.g., positive lymph nodes), dual-targeted therapy with trastuzumab and pertuzumab combined with adjuvant chemotherapy is recommended (the commonly used chemotherapy regimen is anthracycline followed by taxane EC-P, or taxane combined with carboplatin TCb). Pertuzumab is administered once every 3 weeks for 1 year, at a dose of 420 mg (with a first dose of 840 mg). For HER2-positive patients with lymph node-negative breast cancer, when accompanied by other poor prognostic indicators (e.g., Ki67 > 30%, G3, pT_2+_, etc.), the adjuvant trastuzumab and pertuzumab dual-target therapy with chemotherapy can also be recommended. For patients with intermediate or high recurrence risk, especially ER^+^, 1 year of an intensive tyrosine kinase inhibitor such as neratinib after trastuzumab treatment can also be considered [50–55].

Level of evidence: high; level of recommendation: recommended.

(3) For HER2-positive patients not achieving pCR after neoadjuvant therapy, T-DMl (once every 3 weeks, for a total of 14 sessions) can replace trastuzumab. Despite limited evidence, in the case of pCR patients not treated with or with no access to T-DM1, adding adjuvant intensive tyrosine kinase inhibitor (e.g., neratinib) therapy can be considered [50–55].
(4) For patients with concerns about cardiotoxicity, anthracycline-omitting regimens with relatively low cardiotoxicity can be selected: TCbH, TC4H (here C refers to CTX), and wPH (APT trial, weekly paclitaxel plus trastuzumab regimen) [50–55].
(5) Biosimilars of trastuzumab can be used in accordance with the indications on the instruction sheets of these approved drugs in China.

### Clinical guidelines for neoadjuvant therapy of breast cancer

#### Population selection for neoadjuvant therapy for breast cancer

The suitable population for neoadjuvant therapy can be divided into the mandatory population and the preferred population according to the purpose of neoadjuvant therapy. The mandatory population refers to patients who are purported for clinical downstaging and those to be operated on after downstaging (e.g., those with advanced breast cancer that is locally inoperable who have a strong desire for downstaging breast conservation and downstaging axillary conservation). The preferred population refers to those from which in vivo drug susceptibility information can be obtained to guide their later treatments. According to the current evidence, the effect of neoadjuvant therapy with the same regimen and course of treatment is the same as that of adjuvant therapy, but neoadjuvant therapy can enable some patients contraindicated for breast-conserving to undergo breast-conserving therapy and some patients who are inoperable to become eligible for surgery. Patients who do not achieve pCR after neoadjuvant therapy have the opportunity to use dose-intensive treatment regimens to further reduce the risk of recurrence and death. However, some patients (fewer than 5%) may progress during neoadjuvant therapy and even lose the opportunity to undergo surgery. Not all breast cancer patients requiring adjuvant therapy are suited for neoadjuvant therapy. Neoadjuvant therapy can sometimes provide a chance to downstage breast cancer so that the patient can have axilla-preserving surgery that was once impossible. Chinese experts are cautious about this and do not routinely recommend downgrading regional lymph nodes with proven metastases to preserve the axilla as a goal of neoadjuvant therapy [56].

Neoadjuvant treatment for inoperable occult breast cancer is feasible. For patients who need to postpone the operation (e.g., waiting for genetic testing results when developing a surgical plan so that there is time to consider the reconstruction plan) or those who inevitably need to delay the operation (e.g., due to COVID-19 pandemic), neoadjuvant treatment can be given first.

### Implementation of neoadjuvant therapy for breast cancer

#### Preparation before treatment

1. Baseline physical examination of the lesion. Accurately measure the longest diameter of all primary breast lesions and the short diameter of the axillary lymph node.
2. Baseline imaging evaluation. Ultrasound and mammogram examinations are indispensable, and breast MRI examination should be routinely performed for patients who need breast-conserving surgery after downstaging.
3. Blood routine, liver and kidney function, electrocardiogram, chest CT (unenhanced or enhanced), and liver ultrasonography. For patients with locally advanced breast cancer or inflammatory breast cancer, an additional whole-body bone scan and chest CT are recommended. Although brain assessment or positron emission tomography (PET)/CT has certain suggestive significance, it is not a routinely recommended examination item for patients receiving neoadjuvant therapy due to lack of unified imaging evaluation indicators and poor clinical accessibility. Baseline cardiac function tests (e.g., echocardiographic left ventricular ejection fraction) are recommended.
4. Core-needle biopsy (or vacuum-assisted biopsy) must be performed on the primary breast tumor before treatment. The diagnosis of invasive carcinoma or carcinoma in situ (possibly with histological underestimation) accompanied by fine-needle (or core-needle) puncture–confirmed ipsilateral axillary lymph node metastasis must have a clear histological diagnosis and IHC examination (except for occult breast cancer).
5. Whether enlarged regional lymph nodes are breast cancer metastases should be pathologically confirmed through fine-needle (or core-needle) puncture. The area of ​​the patient’s primary lesion is labeled by placing metal marker or making an epidermal tattoo under the guidance of ultrasound to provide a basis for the treatment scope of surgery of the primary lesion.
6. Axillary SLNB can be performed on patients with negative clinical lymph nodes before neoadjuvant treatment, which can provide more information for surgery and systemic treatment.

#### Regimens of neoadjuvant treatment for breast cancer (Table 10 in Appendix)

The neoadjuvant treatment plan should be designed based on the molecular classification of the breast cancer, the availability of drugs, and the conditions of the individual patient. Neoadjuvant treatment options are as follows: chemotherapy with or without targeted therapy (e.g., for HER2-positive patients, add anti-HER2 therapy; for triple-negative patients, add immunotherapy), endocrine therapy with or without targeted therapy (e.g., for HR-positive/HER2-positive patients, add both), and anti-HER2 therapy alone (e.g., for HR-negative/HER2 positive patients) [23, 57–59].

Level of evidence: high; level of recommendation: recommended.

1. For HR-positive/HER2-negative breast cancer patients who need downstaging or breast conservation, it is recommended that adjuvant chemotherapy advance to the neoadjuvant treatment stage. Neoadjuvant endocrine therapy has a similar clinical response rate as neoadjuvant chemotherapy and is a reasonable choice for neoadjuvant therapy. The existing evidence does not support the routine addition of CDK4/6 inhibitors to neoadjuvant endocrine therapy. For postmenopausal patients, AIs are usually used for neoadjuvant endocrine therapy; for premenopausal patients, routine neoadjuvant endocrine therapy is not recommended unless they enter a clinical study or have contraindications to chemotherapy (OPS+AI/fulvestrant is optional). The optimal duration of neoadjuvant endocrine therapy is unclear, but it should generally be continued for 3 to 6 months or until it achieves the best effect [60, 61].

Level of evidence: intermediate; level of recommendation: recommended.

(2) For patients with HER2-positive breast cancer who are to be treated with neoadjuvant therapy, trastuzumab combined with pertuzumab should be used for the neoadjuvant therapy. The preferred chemotherapy combination is taxane combined with carboplatin (TCbHP, PCbHP), while a sequential regimen of anthracycline and taxane is also an option (ECHP-THP). For patients who are intolerant to or unwilling to receive chemotherapy, if they are HR positive/HER2 positive, endocrine therapy combined with anti-HER2 therapy can be considered, and if they are HR negative/HER2 positive, anti-HER2 therapy alone can be considered [60, 61].

Level of evidence: high; level of recommendation: recommended.

(3) For triple-negative breast cancer patients who are to be treated with neoadjuvant therapy, conventional regimens containing anthracyclines and taxanes (EC-T, EC-P) are recommended. Platinum drugs (TCb, PCb or EC-TCb, EC-PCb) can be used as a part of the neoadjuvant treatment regimen for triple-negative patients, but the decision whether to add platinum drugs should weigh the potential benefits and harms, because their addition does not necessarily translate into long-term benefit in disease-free survival. *BRCA1*/*2* pathogenic or suspected pathogenic variants alone are not enough to justify platinum-based therapy. For patients with underlying cardiac diseases, neoadjuvant therapy with taxane + platinum alone can be considered. Although the Keynote522 and IMpassion031 studies suggested that the neoadjuvant addition of PD-1/PD-L1 antibody therapy for early triple-negative breast cancer can improve pCR, it is not routinely recommended to add immune checkpoint inhibitors to neoadjuvant therapy for these patients because they have unknown long-term toxicity and benefits and because the related indications of PD-1/PD-L1 antibodies have not been authorized in China [62–65].

Level of evidence: high; level of recommendation: recommended.

#### Efficacy evaluation and regimen adjustment of neoadjuvant therapy for breast cancer

1. Under normal circumstances, it is recommended to fully evaluate the efficacy before the third cycle of the plan. According to RECIST 1.1, the evaluation results are categorized into the following outcomes: complete response (CR), partial response (PR), stable disease (SD), and progressive disease (PD).
2. The follow-up neoadjuvant treatment plan can be determined (i.e., to implement the established plan or adjust the plan) according to the efficacy evaluation result of neoadjuvant therapy. During the neoadjuvant treatment, attention should be paid to the evaluation and judgment of the early curative effect (2–4 courses). When it is judged to be a significantly increased SD or PD, it is recommended to divide it into two situations: One is inoperable, for which it is recommended to immediately change the neoadjuvant treatment plan empirically and closely evaluate the efficacy; the other is operable, for which surgery should be performed as soon as possible. Particularly for the luminal-type patients, or the triple-negative or HER2-positive patients with poor efficacy after four courses of a standard regimen, the expert group recommends that the patient undergo radical surgery as soon as possible. Some experts also recognize that the neoadjuvant treatment can be changed empirically and closely evaluated, and the latter is still valuable for in vivo drug susceptibility testing [66, 67].
3. For patients with CR, PR, or insignificantly increased SD, it is currently recommended to complete the planned course of neoadjuvant therapy to avoid temporary interruption of neoadjuvant therapy and immediately proceed to surgery in the case of effective treatment. It is recommended that a total of six to eight courses of neoadjuvant chemotherapy ± targeted therapy be done. For patients who have completed the above treatment plan, postoperative adjuvant chemotherapy can be waived; for some patients who have not achieved pCR, intensive treatment can be considered [66, 67].
4. The later adjuvant treatment plan should be determined based on the efficacy evaluation at the end of neoadjuvant therapy. For patients who have not reached pCR, especially triple-negative and HER2-positive patients, intensive adjuvant treatment can be given.

### Local and systemic treatment of breast cancer after downstaging through neoadjuvant therapy

#### Local treatment

1. Breast surgery: Breast-preserving surgery or total mastectomy can be selected according to the patient’s condition.
2. Axillary lymph node surgery: In the case of negative SLNs before neoadjuvant treatment, axillary lymph node evaluation after neoadjuvant treatment can be waived. For patients with axillary lymph node biopsy–confirmed metastasis or SLN metastasis before neoadjuvant treatment, most Chinese experts recommend that axillary dissection should only be replaced by SLNB with caution. For patients with macrometastasis or micrometastasis in axillary SLNB after neoadjuvant treatment, or those of stage T_4_ or N_2/3_ before neoadjuvant treatment, axillary dissection is generally recommended. Refer to the *Clinical Guidelines for Sentinel Lymph Node Biopsy of Breast Cancer* for details.
3. Postoperative adjuvant radiotherapy: Determine whether adjuvant radiotherapy is needed and the range of radiotherapy based on the clinical stage of the tumor before chemotherapy. The range of radiotherapy includes the whole chest wall and the supraclavicular and subclavian areas. In cases in which internal mammary lymph nodes are clinically involved or highly suspected of clinical involvement, radiotherapy of the internal mammary region should be added [17].

Level of evidence: high; level of recommendation: recommended.

#### Systemic treatment

The subsequent adjuvant therapy plan should be determined based on the efficacy evaluation result at the end of neoadjuvant therapy. For patients who have not achieved pCR (who have completed full-course neoadjuvant therapy), especially those with triple-negative breast cancer, an additional six to eight courses of capecitabine therapy can be used (there is not enough evidence for intensive regimens of single-agent platinum drugs or other intravenous chemotherapy). For HER2-positive patients, the intensive T-DM1 adjuvant treatment (once every 3 weeks, for a total of 14 treatments) is preferred. When T-DM1 is not available, intensive TKI-containing regimens can be adopted. When the tumor shows good shrinkage (e.g., shrinkage > 90%, MP=4), the 1-year plan of trastuzumab combined with pertuzumab can be started. Regardless of whether pCR is achieved, the ExteNET trial showed that the 1-year extension of neratinib treatment in selected populations further reduces the risk of relapse. For HR-positive patients, endocrine therapy should be adopted. Whether intensive endocrine therapy is needed and, if so, the manner of intensification can be determined mainly by evaluating the patient’s status before neoadjuvant therapy [66].

Level of evidence: high; level of recommendation: recommended.

## Part 5: Treatment: Advanced Breast Cancer

### Clinical guideline for systemic salvage therapy for advanced breast cancer

Advanced breast cancer, including recurrent and metastatic breast cancer, is an incurable disease. The main purpose of advanced breast cancer treatment is to relieve symptoms, improve quality of life, and prolong the patient’s life. If possible, recurrent or metastatic lesions, especially solitary lesions, should be biopsied before treatment to confirm the diagnosis and reassess the tumor’s ER, PR, and HER2 status. The value of local treatments (e.g., surgery and radiotherapy) in de novo stage IV breast cancer is unclear. Only when the systemic drug therapy achieves a good effect should palliative local therapy be considered so that the effect of systemic therapy will be consolidated. For patients with local or regional relapse but no distant metastasis, radical treatment should be performed if they are deemed suitable for radical local treatment after comprehensive evaluation. For example, patients with relapse after breast-conserving therapy can be treated with total mastectomy; those with recurrence of chest wall or regional lymph nodes can be treated by resecting the involved site and lymph nodes; those who have not received radiotherapy before can be treated with local radiotherapy; adjuvant chemotherapy again (mainly in HR-negative patients), targeted therapy, and endocrine therapy may also have certain value.

### Clinical guidelines for endocrine therapy of advanced breast cancer

#### Population selection for endocrine therapy of advanced breast cancer

1. Recurrent or metastatic ER- and/or PR-positive breast cancer. Endocrine therapy may be considered in patients with unknown receptors but clinically showing a slow disease course.
2. Patients without visceral crisis. Visceral crisis refers to abnormal functioning of several organs, confirmed by symptoms, signs, laboratory tests, and rapid disease progression. It is not simply the presence of visceral metastases, but a critical visceral condition requiring rapid and effective treatment to control disease progression, especially if the window of opportunity for chemotherapy is closing with the progression. Endocrine therapy is considered more appropriate for asymptomatic visceral metastases and/or osteochondral metastases.
3. The time from the start of adjuvant endocrine therapy to relapse is relatively long (generally more than 2 years), and this time limit can be appropriately extended when combined with certain targeted drugs.

#### Conversation with the advanced breast cancer patient before endocrine therapy

1. Systemic therapy for recurrent or stage IV breast cancer aims mainly to prolong life and improve quality of life rather than cure the disease, so treatments with less toxicity are preferred. The disease control rate and progression-free survival (PFS) after endocrine therapy combined with targeted therapy are noninferior to or are even superior to those of chemotherapy. There are several options for endocrine therapy, which can be performed sequentially to maximally prolong the time before a patient starts chemotherapy.
2. Education of patients on adverse reactions to endocrine therapy.

#### Concepts related to endocrine therapy for advanced breast cancer

1. Primary endocrine resistance refers to a relapse or metastasis of early-stage breast cancer within 2 years of adjuvant endocrine therapy or disease progression within 6 months of endocrine therapy for metastatic breast cancer.
2. Secondary endocrine resistance refers to a relapse or metastasis that occurs after 2 years of endocrine therapy and during or within the first year after completion of adjuvant endocrine therapy for early-stage breast cancer or disease progression after more than 6 months of endocrine therapy for metastatic breast cancer.
3. Endocrine sensitivity refers to de novo stage IV breast cancer that was never treated with endocrine therapy or a relapse or metastasis at least 2 years after completing postoperative adjuvant endocrine therapy for early-stage breast cancer.
4. First-line and second-line endocrine therapies usually correspond to the first and second endocrine therapy regimens, respectively, received after relapse and metastasis. Since the definition applies to subsequent treatment decisions, it is recommended that the first-line endocrine therapy be defined as the first endocrine therapy regimen for endocrine-sensitive patients after relapse or metastasis, and the second-line endocrine therapy be defined as the endocrine rescue regimen received by patients with primary or secondary endocrine resistance after relapse or metastasis [9].

#### Drugs for endocrine therapy of advanced breast cancer

1. Recommendations for endocrine therapy for postmenopausal patients: AIs include nonsteroids (anastrozole and letrozole), steroids (exemestane), ER modulators (tamoxifen, toremifene), ER downregulators (fulvestrant), progesterones (megestrol, medroxyprogesterone), androgens (fluoxymesterone), and high-dose estrogens (ethinyl estradiol) [68–70].
2. Recommendations for endocrine therapy for premenopausal patients: On the basis of ovarian function suppression (mainly through luteinizing hormone–releasing hormone analogs and surgical castration), premenopausal breast cancer patients can be treated the same way the postmenopausal breast cancer patients are treated. For those without ovarian function suppression, ER modulators (tamoxifen, toremifene), progesterones (megestrol, medroxyprogesterone), androgens (fluoxymesterone), and high-dose estrogens (ethinyl estradiol) can be considered [68–70].
3. For both premenopausal and postmenopausal patients, on the basis of endocrine therapy, combined targeted therapy (e.g., CDK4/6 inhibitors, mTOR inhibitors, histone deacetylase inhibitors, etc.—PI3Kα inhibitors have not been marketed in China yet) can be considered [71].

#### Selection of first-line endocrine therapy for advanced breast cancer and precautions

1. AIs combined with CDK4/6 inhibitors (palbociclib, abemaciclib, ribociclib) are the preferred first-line endocrine therapy for HR-positive/HER2-negative postmenopausal or premenopausal breast cancer patients after medical castration. Combinations with CDK4/6 inhibitors can significantly improve the patient’s PFS, and some studies indicated that it can even improve OS.[68–70].

Level of evidence: high; level of recommendation: recommended

(2) Fulvestrant (± ovarian function suppression (OFS)) combined with CDK4/6 inhibitor is not preferred and was not superior to AI (± OFS) combined with CDK4/6 inhibitor in the PARSIFAL study. Tamoxifen + OFS combined with CDK 4/6 inhibitor was beneficial in terms of PFS and OS in the MONALEESA-7 study and can be selected under certain circumstances [68–70, 72].

Level of evidence: intermediate; level of recommendation: recommended

(3) When CDK4/6 inhibitors are not available, single-agent endocrine therapy can be an alternative. For postmenopausal patients, fulvestrant, AI, ER modulators (tamoxifen, toremifene) can be used. For premenopausal patients, OFS combined with fulvestrant, OFS combined with AI, OFS combined with ER modulators, and ER modulators alone can be selected [71].

Level of evidence: high; level of recommendation: recommended

(4) After treatment with ovarian function inhibitors, premenopausal patients can be managed the same way as postmenopausal patients.

#### Selection of second-line endocrine therapy for advanced breast cancer and precautions

If the first-line endocrine therapy fails, patients without visceral crisis can still choose second-line endocrine therapy ± targeted therapy. It is not recommended to repeatedly use endocrine drugs with proven resistance to adjuvant or first-line therapy. After treatment with ovarian function inhibitors, premenopausal patients can be managed the same way as postmenopausal patients are managed.

1. For patients who have not used CDK4/6 inhibitors: ① Fulvestrant combined with CDK4/6 inhibitor (palbociclib, abemaciclib, ribociclib) is the preferred regimen of second-line endocrine therapy for HR-positive/HER2-negative postmenopausal or premenopausal breast cancer patients after medical castration. The addition of CDK4/6 inhibitor can significantly improve the patient’s PFS and OS. For patients with primary endocrine resistance, the evidence for the benefit of fulvestrant in combination with the specific CDK4/6 inhibitor abemaciclib is relatively strong. ② Steroidal/nonsteroidal AI (± OFS) or tamoxifen (± OFS) combined with CDK 4/6 inhibitor may also be used in certain circumstances. For patients who have already taken a CDK4/6 inhibitor, there is not enough evidence to support cross-line therapy with a CDK4/6 inhibitor [71].

Level of evidence: high; level of recommendation: recommended

(2) The mTOR inhibitor everolimus and the histone deacetylase inhibitor chidamide can be considered in combination with endocrine therapy as second-line therapy. There is evidence for the benefit of the PI3Kα inhibitor alpelisib when combined with endocrine therapy in patients with PI3Kα mutation (detected in tumor tissue or peripheral blood ctDNA), which has been already indicated in the US and EU but not yet in China [9].

Level of evidence: high; level of recommendation: recommended

(3) For patients with primary endocrine resistance, if the above small-molecule targeted drugs in those combinations are not available, chemotherapy can be used for rescue treatment.

Level of evidence: intermediate; level of recommendation: recommended

### Clinical guidelines for chemotherapy ± targeted therapy of advanced breast cancer

#### Population selection of chemotherapy ± targeted therapy of advanced breast cancer

Chemotherapy ± targeted therapy can be considered if any of the following factors is present:

1. Hormone receptor negativity or low hormone receptor expression.
2. With visceral crisis or symptomatic visceral metastasis.
3. Hormone receptor positivity but with proven resistance to endocrine therapy (especially primary resistance).

### Conversation with advanced breast cancer patient before chemotherapy ± targeted therapy

1. The purpose of chemotherapy ± targeted therapy is to improve quality of life and prolong PFS and OS.
2. Education for patients with adverse reactions to chemotherapy ± targeted therapy.

### Preparation for chemotherapy ± targeted therapy for advanced breast cancer

1. Before the first chemotherapy, routine blood tests, liver and kidney function tests, and electrocardiogram should be performed. Blood routine tests should be performed before each chemotherapy subsequently, and patients with abnormal liver and kidney function should be monitored continuously. For those using anthracyclines, ECG and left ventricular ejection fraction (LVEF) should also be performed, and those with abnormal ECG and LVEF should be continuously monitored.
2. Women of childbearing age should have a negative pregnancy test and be prescribed a contraceptive.
3. The patient must sign the informed consent form for antitumor treatment.

### Selection of chemotherapy ± targeted therapy for HER2-negative advanced breast cancer and precautions (Table 11 in Appendix)

1. The recommended preferred chemotherapy regimens include single-agent sequential chemotherapy or combination chemotherapy. Sequential use of a single agent is preferred since it can ensure treatment tolerance and quality of life. Compared with single-agent chemotherapy, combination chemotherapy generally yields better outcomes in terms of response rate and PFS but is more toxic and has failed to demonstrate an OS benefit. Combination chemotherapy is an option for patients who need fast tumor shrinkage or fast symptom relief [68, 73–78].

Level of evidence: high; level of recommendation: recommended

(2) Anthracycline (taxane) treatment failure is commonly defined as disease progression during rescue chemotherapy with anthracyclines (taxanes) or relapse and metastasis within 12 months after the end of adjuvant therapy. For patients who have had failed therapy with anthracyclines, single-agent or combination regimens based on taxanes (e.g., paclitaxel, docetaxel, nab-paclitaxel) are usually preferred; for those have had failed therapy with both anthracyclines and taxanes, there is no standard chemotherapy regimen, and other single-agent or combination regimens can be considered [68, 73–77].
(3) Commonly used single-agent drugs include anthracyclines (e.g., doxorubicin, epirubicin, pirarubicin, PEGylated liposomal doxorubicin), taxanes (e.g., paclitaxel, docetaxel, nab-paclitaxel), antimetabolites (e.g., capecitabine, gemcitabine), nontaxane microtubule inhibitors (e.g., vinorelbine, eribulin, utidelone), platinum compounds (e.g., cisplatin, carboplatin), and the topoisomerase inhibitor etoposide [68, 73–77].
(4) Combination chemotherapy regimens are diverse and are formulated mainly based on the previous evidence, the interaction between the combination drugs, the toxicity spectrum of the combination drugs, and the status of the individual patient. Combining three or more chemotherapy drugs is not recommended. For triple-negative breast cancer, the GP regimen (gemcitabine combined with cisplatin, especially for patients with defects in *BRCA1*/*2* and other homologous recombination repair genes), the GC regimen (gemcitabine combined with carboplatin), the AP regimen (nab-paclitaxel combined with cisplatin/carboplatin), and the PC regimen (other taxanes combined with carboplatin/cisplatin) can be selected [68, 73–77].

Level of evidence: high; level of recommendation: recommended

(5) Single-agent or combination chemotherapy can be combined with targeted therapy under the support of evidence medicine. According to the IMpassion130 and Keynote355 studies, nab-paclitaxel + atezolizumab (when PD-L1 IC is positive), nab-paclitaxel/paclitaxel/GC + pembrolizumab (when PD-L1 CPS is ≥ 10:00) can be tried, but since PD-1/L1 antibody therapy has not yet reached the corresponding indications, patients should be carefully selected in clinical practice. Chemotherapy combined with the anti-angiogenic drug bevacizumab can benefit in terms of disease progression and PFS, but it does not prolong OS, so it is not recommended for routine use but can be given carefully to patients who urgently need tumor or symptom control [68, 73–77].
(6) In combined chemotherapy, whether to use a continuous method or to discontinue the drug or maintain the treatment after 4-8 cycles of treatment needs to be decided by weighing the efficacy, adverse drug reactions, and the patient’s quality of life. For patients in whom combination chemotherapy is effective but cannot tolerate combination chemotherapy or are unwilling to continue it, maintenance therapy can be considered, and chemotherapy with a single-agent selected from the original combination regimen (e.g., oral capecitabine, vinorelbine) can be considered. For those with positive hormone receptors, maintenance therapy with endocrine ± targeted therapy can also be considered [68, 73–77].
(7) For patients with *BRCA1*/*2* germline pathogenic variants or suspected pathogenic *BRCA1*/*2* mutations, poly (ADP-ribose) polymerase (PARP) inhibitors (olaparib/talazoparib) can be selected for treatment, one of which, olaparib, has been marketed in China but has not yet been approved for corresponding indications. These patients can also consider participating in related clinical studies [74].
(8) For triple-negative breast cancer, sacituzumab govitecan-hziy is an important targeted therapy option and has been approved by the US FDA but is still under review in China [9].

### Clinical guidelines for the treatment of HER2-positive advanced breast cancer

#### Population selection for anti-HER2 therapy for advanced breast cancer

In patients with HER2-positive recurrent or metastatic breast cancer, if the HER2 test results are inconsistent between the primary tumor and metastases or between metastases at different times, then the test result of the most recent metastasis test should be adopted. Considering the spatiotemporal heterogeneity of HER2 status, it should not completely exclude continuous anti-HER2 therapy, selected with caution, while continuously monitoring the response even if the most recent metastasis test shows HER2 negativity.

#### Precautions for the use of anti-HER2 mAbs

1. Trastuzumab and pertuzumab: LVEF < 50% before treatment. Baseline cardiac function should be assessed before application, and they should not be given to people at high risk of cardiovascular events.
2. Simultaneous use of drugs with synergistic damaging effects such as anthracyclines should be avoided.
3. Cardiac function should be regularly assessed during treatment. If LVEF has decreased by ≥ 15% from the baseline or is lower than the normal range and has decreased by ≥ 10%, anti-HER2 therapy should be suspended, and LVEF should be reexamined within 3-4 weeks to determine whether anti-HER2 therapy can be continued.
4. Trastuzumab emtansine (T-DM1): Standardized monitoring of platelets should be performed at baseline and during the medication period. If thrombocytopenia occurs, the dose should be reduced, or the medication should be discontinued promptly. When thrombocytopenia of grade ≥ 2 occurs, it should alert the clinician to the possibility of persistent thrombocytopenia. If the conventional platelet-raising therapy is not effective, specialists should be consulted promptly to handle the situation.

### Conversation with advanced breast cancer patient before anti-HER2 therapy

1. Patients with HER2-positive breast cancer should be adequately informed of the benefits of timely anti-HER2 therapy. Anti-HER2 drugs mainly include trastuzumab and its biosimilars, pertuzumab, inetetamab, margetuximab, lapatinib, pyrotinib, neratinib, tucatinib, T-DM1, DS8201, etc.
2. The monoclonal antibody trastuzumab and similar-acting antibodies such as pertuzumab and inetetamab have good overall safety but may affect the cardiac ejection function and increase the probability of congestive heart failure. TKI drugs (lapatinib, pyrotinib, neratinib, tucatinib) have gastrointestinal reactions such as diarrhea. T-DM1 has a risk of thrombocytopenia. DS8201 has a risk of interstitial lung disease. When using the above drugs, it is necessary to follow the doctor’s advice with regular follow-up monitoring (e.g., reexamination of LVEF in every 3 months when using monoclonal antibodies).

### Preparation before anti-HER2 therapy for advanced breast cancer

1. Accurate HER2 testing. If necessary, a wax block or unstained paraffin section should be sent to the pathology department of a hospital widely recognized in China for review. Rebiopsy of metastases should be performed if possible so that the HER2 status of the metastases can be determined.
2. Cardiac function examination (cardiac ultrasonography or radionuclide scan, of which the former is more commonly used).
3. Signing of the informed consent form for antitumor treatment.

### Selection of anti-HER2 therapy for advanced breast cancer and precautions

Continuous anti-HER2 therapy is an important treatment principle for HER2-positive advanced breast cancer.

1. For patients who have never used trastuzumab or who meet the conditions for reuse of trastuzumab (relapse and metastasis more than one year after the end of adjuvant trastuzumab therapy), trastuzumab ± pertuzumab–based first-line therapy, preferably in combination with taxanes, is preferred. The first-line therapy of taxanes combined with the dual-targeting trastuzumab + pertuzumab can prolong PFS and OS over taxanes + trastuzumab.

Level of evidence: high; level of recommendation: recommended

(2) For patients who have not seen success with trastuzumab ± pertuzumab, single-agent T-DM1 can prolong PFS and OS; pyrotinib (or neratinib) combined with capecitabine is superior to lapatinib combined with capecitabine in terms of PFS. Combinations of two targeted agents alone (e.g., lapatinib + trastuzumab) have also improved OS. The expert group recommends that for patients with recurrent and metastatic breast cancer 1 year after the end of adjuvant pertuzumab + trastuzumab therapy, the optional first-line rescue treatment regimens include pyrotinib + capecitabine, T-DM1 or pertuzumab + trastuzumab + docetaxel, etc. Margetuximab, tucatinib, and DS8201a have shown certain value in clinical studies after multiline treatment, but they still have not been approved in China, so they need to be selected with caution.

Level of evidence: high; level of recommendation: recommended

(3) Trastuzumab is allowed in cross-line therapy.

Level of evidence: high; level of recommendation: recommended

(4) For HR-positive/HER2-positive patients who cannot tolerate or refuse chemotherapy or are undergoing maintenance therapy after chemotherapy, endocrine therapy + anti-HER2 (single-target or dual-target) therapy can be used, although there is no clear evidence that it can improve OS [47].

Level of evidence: intermediate; level of recommendation: recommended

(5) Biosimilars are therapeutic biological products that are nearly identical to the original product and have similar quality, safety, and efficacy. Trastuzumab biosimilars have been approved in China and can be appropriately extrapolated for indications related to HER2-positive breast cancer.
(6) For patients with brain metastases, TKI drugs are the preferred options.
(7) If multiline anti-HER2 treatment fails and further treatment cannot be obtained, it is recommended that the patient participate in a clinical study.

## Clinical guidelines for palliative care in end-stage breast cancer

Palliative care is a clinical discipline that prevents and relieves physical and psychological distress through early identification, proactive assessment, pain management, and treatment of other disease-related symptoms, including physical, psychosocial, and spiritual troubles, and improves the quality of life of patients with life-threatening illnesses and their family members [79].

### Applicable population

1. Those with uncontrolled tumor-related symptoms (e.g., pain, dyspnea, anorexia, cachexia, nausea and vomiting, etc.).
2. Those with moderate to severe physical and psychological problems related to their cancer diagnosis and treatment.
3. Those with serious concomitant diseases, and mental and psychosocial conditions.
4. Those with an expected survival time ≤ 6 months.
5. Patients and their families who have the desire to understand the disease development process and participate in treatment decision-making.
6. Patients and their families who have a need for palliative care.

### Conversation with end-stage breast cancer patient before palliative care

1. Communicate with patients and their families so that they can understand the natural course and prognosis of the disease, the value of antitumor therapy, possible adverse reactions and complications, and the nature and methods of palliative treatment.
2. Understand the expectations and needs of patients and their families for palliative care, make corresponding treatment decisions, and develop specific measures.
3. Repeatedly communicate with patients and their families during the treatment process to promptly understand their changes in treatment expectations and needs.

### Main measures

1. Provide clinical medical services for the control of pain and other distressful symptoms so that patients can maximally relieve the pain.
2. Maintain and respect life and treat death as a normal process. Do not advocate abandonment of treatment or euthanasia, nor overtreatment. Neither deliberately accelerate death nor deliberately delay it.
3. Unify the patient’s spirit, psyche, and soul for palliative care.
4. Provide a support system to help the patient live as positively as possible until death. Meanwhile, help the patient’s family to properly handle the patient’s disease process. Use teamwork to meet the overall needs of patients and their families, including bereavement services and counseling.
5. Palliative care is also applicable to the early and middle stages of the disease process. There, the main purpose is still to relieve the patient’s physical and mental distress and improve the quality of life.

### Control of tumor-related symptoms

#### Pain

The pain management for advanced cancer should follow the principle of three-step treatment. The so-called three-step ladder for cancer pain is to select the appropriate analgesics according to the pain degree of the patient after a correct assessment of the nature and cause of the pain: nonopioid analgesics ± adjuvant drugs for patients with mild pain; low-dose strong opioids ± nonopioid analgesics ± adjuvant drugs for patients with moderate pain; strong opioids ± nonopioid analgesics ± adjuvant drugs for patients with severe pain [80].

1. Ladder-based medication: Ladder-based medication means that analgesic drugs should be chosen based on the degree of pain of the patient from mild to severe, and analgesic drugs of the same strength should be selected in sequence. For severe pain, it can start with strong opioids so that the pain can be quickly relieved and symptoms eased.
2. Time-based medication: Time-based medication means analgesics are administered regularly at prescribed intervals, in which controlled-release dosage forms are mostly used under steady state conditions. For each analgesic, dose titration from low to high is performed to control the pain to find the best dose. If the patient has sudden severe pain while using an analgesic, an additional dose on top of the original dose can be given promptly to relieve the pain, and the total dose for the patient can be retitrated in the future.
3. Oral or noninvasive medication: Noninvasive medication and oral administration should be preferred. Alternative methods of administration may be used in cases where oral administration is not possible or causes excessive adverse reactions.
4. Individualized medication: The use of medicines needs to be varied from person to person according to the circumstances.
5. Pay attention to details: Closely monitor patients taking analgesics, observe the degree of pain relief and adverse reactions, and take necessary measures in a timely manner. With pain control and symptom relief, the dose can be gradually reduced in some patients to achieve optimal treatment.
6. Cancer pain management should achieve the “4A” goal: optimize analgesia, optimize activities of daily living, minimize adverse effects, and avoid aberrant drug taking.
7. To achieve the “4A” goal, the guidelines in recent years have classified the low-dose step-3 drugs (e.g., morphine with a daily dose of ≤ 30 mg and oxycodone with a daily dose of ≤ 20 mg) into step 2 of the ladder, and clinically, low-dose step-3 analgesics can be used to manage moderate cancer pain.

Adverse reactions and management of narcotic analgesics include:

1. In general, opioids are safe and effective for cancer pain, but patients who require high doses of narcotic analgesics or those who use narcotic analgesics for a long time may develop some symptoms such as constipation, lethargy, and urinary retention. Other symptoms include toxic metabolite accumulation resulting in poisoning, with symptoms including refractory nausea, lethargy, and itching, as well as neurotoxic symptoms including hallucinations, delirium, muscle tremors, and paresthesias, causing respiratory depression when severe.
2. The ways of treating and preventing these adverse reactions include adequate hydration and changes in the type of narcotic analgesic, discontinuation of other drugs that aggravate adverse reactions, prophylactic treatment of anticipated adverse reactions, symptomatic treatment of symptoms that have already manifested, and the use of antidotal antagonists.
3. Be cautious when treating patients with organ insufficiency, especially liver or kidney insufficiency. The dose of narcotic analgesics should be reduced to avoid possible accumulation of metabolites that can cause damage to the body.

Tolerance to and dependence on narcotic analgesics:

1. Tolerance to narcotic analgesics: The dose of narcotic analgesics must be increased, on the one hand, due to the disease progression–caused aggravation of pain from cancer patient, and on the other hand, due to the development of drug resistance by the patient, necessitating a higher dose of analgesic to achieve the same analgesic effect. The mechanism of this normal physiological phenomenon is likely that the level of narcotic analgesic receptors changes due to changes in metabolites.
2. Physiological dependence: For patients who use narcotic analgesics for a long time, physiological dependence is a common and normal pharmacological reaction. Patients may manifest withdrawal symptoms (e.g., restlessness, tremors, fever, sweating, dilated pupils, rapid heartbeat, muscle and abdominal spasms) when the use of narcotic analgesics is suddenly stopped or the dose is suddenly reduced. At this time, if the narcotic analgesic needs to be reduced or stopped, it must be gradually reduced at a rate of 10% to 20% per day.
3. Psychological dependence (addiction): Psychological dependence (addiction) is a psychopathic obsessive-compulsive disorder resulting from the use of a substance, leading to physical, psychological, and sociological harm to the user. Moreover, even with the harm, the user will continue to use the drug compulsively. In fact, cancer patients with no history of alcohol or drug dependence rarely develop psychological addiction when narcotic analgesics are appropriately used.

#### Anorexia and cachexia

End-stage patients often manifest anorexia and malnutrition, also known as anorexia-cachexia syndrome. It is mainly caused by tumor-induced metabolic dysfunction, including abnormal secretion of cytokines, metabolic disorders of insulin and adrenal corticosteroids, immunosuppression, increased fat and protein catabolism, etc., and may also stem from the influence of tumor treatment or psychological factors. Clinical manifestations include significant weight loss, muscle atrophy, anorexia, fatigue, dysgeusia, anemia, hypoalbuminemia, edema, bedsores, and listlessness.

The treatment principle is mainly correction of metabolic abnormalities, appropriate nutritional support, and strengthening psychological support and care. In specific clinical implementations, it is necessary to make sure the patient has enough nutrients and energy to achieve the purpose of nutritional support while avoiding too much nutrients and energy, especially for elderly patients and those with organ dysfunction.

A certain amount of nutrients and energy should be given according to the laboratory test indicators and the intake and output amounts. It is recommended that enteral nutrition be mainly given, while appropriate intravenous nutrition be given in the case of abnormal water and electrolyte balance or insufficient enteral nutrition. In addition, corticosteroids, progesterones (megestrol, medroxyprogesterone), and gastric motility drugs can be appropriately used as adjuvant therapies.

#### Nausea and vomiting

1. Identify the cause of vomiting, such as treatment-related vomiting (e.g., chemotherapy, radiotherapy, etc.) and disease-related vomiting (e.g., brain metastasis, gastrointestinal obstruction, etc.).
2. Treat the cause, such as by administering preventive antiemetic drugs before radiotherapy and chemotherapy, dehydration for patients with brain metastases, and gastrointestinal decompression for patients with gastrointestinal obstruction.
3. For nonspecific nausea and vomiting, especially caused by anxiety, dopamine receptor antagonists or benzodiazepines can be used.
4. Intractable nausea and vomiting can be treated with drugs administered intravenously or subcutaneously, and the dose of dopamine receptor antagonists can be titrated to the maximum benefit and highest tolerable level. If nausea persists, serotonin receptor antagonists and/or anticholinergics and/or antihistamines, glucocorticoids, tranquillizers, or even cannabinoids can be added. Acupuncture and sedatives may also be considered.
5. Be aware that severe vomiting may cause upper gastrointestinal bleeding, and the electrolyte balance should be monitored.

#### Fatigue

Fatigue is a very common and serious symptom of advanced cancer. It manifests in almost all patients with advanced cancer, especially as the disease progresses to the end stage. It can reduce the patient’s psychological and physical endurance and prevent them from living normally. The clinical manifestations of fatigue include physical insufficiency, tiredness, lethargy, and mental decline, which seriously affect the patient’s quality of life. Fatigue may also exacerbate other symptoms such as pain, depression, and sleep disorders.

Fatigue is mostly caused by malnutrition, cachexia, drugs, radiotherapy, pain, mood and sleep disorders, water–electrolyte imbalance (e.g., hypokalemia, hyponatremia, dehydration, etc.), hypoxia, metabolic disorders (e.g., nutrient depletion by tumor, blood sugar changes, acidosis), low hemogram (e.g., anemia), heart, liver or kidney failure, endocrine disorders, or infections.

Treatment of fatigue generally targets the cause (e.g., analgesia; anti-infection; protection of heart, liver, and kidney functions) and corrects deficiencies (e.g., water and electrolytes, blood sugar, red blood cells, white blood cells, platelets, blood oxygen). Some adrenal corticosteroids (e.g., dexamethasone), progestins (e.g., megestrol, medroxyprogesterone), or psychostimulants (e.g., methylphenidate) can be added in supportive treatment.

#### Coma

Coma is a clinical manifestation of severe brain dysfunction, with persistent loss of consciousness despite the presence of vital signs, very common in end-stage patients short expected survival. The degree of coma can be light or deep, according to the presence or absence of the withdrawal response to pain and the presence or absence of the pupillary reflex and corneal reflex.

Clinical manifestations: ① When in light coma, patients lose most of their consciousness and have no voluntary activities. They can have painful expressions and limb withdrawal reactions under strong stimulation and defensive reflexes under pain stimulation. They still have corneal reflexes, eye movements, swallowing reflexes, frequent pathological reflexes, and likely urinary incontinence or urinary retention. ② When in deep coma, patients completely lose consciousness as well as all deep and shallow reflexes, with flaccid paralysis in limbs and only breathing and circulatory functions.

Common causes of coma in tumor patients are craniocerebral space–occupying lesions, invasion of the central nervous system by malignant tumors, high fever, infection, metabolic disorders, electrolyte imbalance, and cerebral hemorrhage.

Coma in most cancer patients indicates that the disease has progressed to a very late stage, with a very poor prognosis, so the treatment should be moderate. ① Etiological treatment: For craniocerebral space–occupying lesions and malignant tumors that invade the central nervous system, treatment of dehydration and hormonal imbalances should be performed. For high fever, infection, metabolic disorders, electrolyte imbalances, and cerebral hemorrhage, supportive treatment targeting the cause should be performed. For light coma, local palliative radiotherapy can be done. ② Supportive care: The purpose is to ensure adequate blood sugar and nutrition, maintain venous access, correct any acid–base imbalance, and maintain the water–electrolyte balance. ③ Strengthen nursing care: Try to make the patient’s head turn to one side, keep the patient warm, put in an indwelling catheter, keep the patient’s skin dry and clean, and take care to prevent and treat bedsores. It is necessary to keep the airway unobstructed and supplement oxygen in the case of hypoxia or dyspnea, and select appropriate antibiotics in the case of infection. Xingnaojing can be appropriately used when necessary. When in a deep coma, the patient cannot feel pain, so if the family agrees or requests, no further treatment can be performed.

## Part 6: Follow-up, Rehabilitation, and Traditional Chinese Medicine (TCM) Treatment of Breast Cancer Patients

### Follow-up and evaluation

#### Frequency of follow-up

The frequency of follow-up of breast cancer patient needs to be decided based on the risk of relapse. The recommendations are as follows:

1. Within 2 years after surgery, once every 3 months.
2. Within 3 to 5 years after surgery, once every 6 months.
3. More than 5 years after surgery, once a year for life.

If there is any abnormal situation, the patient should seek medical treatment promptly without adhering to a fixed schedule.

Level of evidence: low; recommendation level: recommended

#### Follow-up evaluation items

The specific contents of the follow-up evaluation items are given in Table 7.

**Table 7:** Follow-up inspection items

Routine examination item	Time and remarks on examination, medical history inquiry, and physical examination
Ultrasound of liver, breast area, and lymphatic drainage area	According to postoperative follow-up frequency
Laboratory tests such as blood routine, liver and kidney function, and blood lipids	According to postoperative follow-up frequency
Mammography and chest CT	According to postoperative follow-up frequency
If the patient has received radiotherapy, start this examination 6 to 12 months after the end of radiotherapy	Once a year; if any abnormality is found, the patient can be reexamined soon.
Bone scan	If suggestive symptoms are present to exclude bone metastases, choose as appropriate
Breast MRI	Optional for patients undergoing breast-conserving surgery, or to supplement other imaging examinations
Gynecological examination and gynecological ultrasound if: • Taking tamoxifen without surgical removal of the uterus and ovaries	Once every 3-6 months
Bone mineral density (BMD) test if: • Postmenopausal or taking third-generation AI	Once a year after baseline examination

Level of evidence: low; recommendation level: recommended

#### Follow-up evaluation items

##### Evaluation of upper limb function

1. Range of motion of upper limbs: It should be back to normal within 1 to 2 months of breast cancer surgery. If movement is limited, intensive functional exercise or further medical treatment is required.
2. Lymphedema of the affected limb: There are many methods for evaluating lymphedema in breast cancer patients undergoing axillary surgery. The clinical judgment is mainly made by asking about the patients’ subjective feelings or by physical examination and measuring multisegment arm circumferences. Generally, if the circumference of the upper limb on the affected side is longer than that of the contralateral upper limb by less than 3 cm, it indicates mild edema; 3-5 cm, moderate edema; and more than 5 cm, severe edema.

Level of evidence: intermediate; level of recommendation: recommended

#### Risk assessment of concurrent diseases

1. Risk assessment of cardiovascular and cerebrovascular events:
  ② Dyslipidemia: Patients receiving endocrine therapy should be evaluated for blood lipid status to determine whether they have dyslipidemia
2. Risk assessment of fracture events: Patients taking third-generation AIs or who have undergone ovarian castration should undergo BMD testing and fracture risk assessment before drug use and at the annual follow-up.

Level of evidence: intermediate; level of recommendation: recommended

#### Lifestyle assessment

1. Weight assessment

At the first follow-up, the patient’s height and weight should be measured. Body weight should be measured at each follow-up. Each time, the patient’s body mass index (BMI) needs to be calculated and is then evaluated as low, normal, overweight, or obese according to *The Guidelines for Prevention and Control of Overweight and Obesity in Chinese Adults*.

(2) Nutrition and exercise

The patient’s daily food intake should be asked about. It is recommended that the 24-hour dietary recall be used to continuously record the dietary details of the last 3 days. Whether the patient’s food intake and main nutrients meet the recommended amounts and whether the patient’s dietary structure is reasonable should be evaluated.

Patients’ daily physical activities should also be asked about, e.g., whether they regularly perform physical exercises such as brisk walking, jogging, dancing, and swimming, and if they do, the frequency and time should be recorded.

(3) Other items

Whether patients smoke or breathe second-hand smoke and whether they drink alcohol should be asked about, and if they do, the frequency and quantity should be recorded. Whether patients use health products or dietary supplements should be asked about, and if they do, the product names and use frequencies should be asked about.

Level of evidence: intermediate; level of recommendation: recommended

### Psychological and social support assessment

Negative emotions of breast cancer patients mainly include low self-esteem, anxiety, and depression. During follow-up, the patient’s psychological status and social support status should be assessed through inquiry and/or validated scales.

Level of evidence: low; recommendation level: recommended

### Sex life and fertility assessment

Breast cancer treatment and treatment-induced adverse reactions (e.g., self-image change after mastectomy, early menopause symptoms, etc.) can affect the patient’s sex life to a great extent, and the persistence or adverse reactions to treatment will also affect the fertility recovery of breast cancer patients of reproductive age. Therefore, it is necessary to assess and follow up the patient’s sex life and reproductive needs in the form of interviews and/or questionnaires.

Level of evidence: low; recommendation level: recommended

## Clinical management and rehabilitation guidance

### Rehabilitation of affected limbs

#### Step-by-step functional exercise of the arm on the affected side

Step-by-step method: ① 1-2 days after surgery, ask the patient to practice making a fist, extending their fingers, and flexing their wrist; ② 3-4 days after surgery, ask the patient to practice extending and flexing their forearms; ③ 5-7 days after surgery, ask the patient to touch the opposite shoulder and ipsilateral ear with the hand on the affected side; ④ 8-10 days after surgery, ask the patient to practice shoulder joint elevation, straightening, and flexion to 90°; ⑤ 10 days after surgery, ask the patient to use their shoulder joint in wall climbing and equipment exercises. Within 1-2 months of surgery, the function of the shoulder joint on the affected side can usually return to its preoperative state or equal the opposite-shoulder function.

The requirements for functional exercise are as follows: within 2 weeks after surgery, the patient can straighten the upper arm of the affected side, raised it over the head, and touch the ear on the other side. Functional exercise should be continued after meeting the above requirements.

Shoulder joint abduction should be limited in the first 7 days after surgery (especially before the removal of an axillary drainage tube). Patients with severe flap necrosis should avoid large movements within 2 weeks after surgery. The frequency of exercise and the movement range of the shoulder joint should be reduced (abduction should be limited) if the subcutaneous fluid or drainage exceeds 50 mL 1 week after surgery. After skin grafting and latissimus dorsi flap breast reconstruction, shoulder joint movements should be postponed, and excessive exercise in the early postoperative period should be avoided.

Level of evidence: low; recommendation level: recommended

#### Prevention of upper extremity lymphedema

1. Prevention of infection: The patient should keep the skin on the affected side clean. Invasive operations on the arm of the affected limb, such as blood drawing, infusion, etc., should be avoided. It is advised that the patient wear loose gloves when washing to avoid prolonged contact with irritating detergents. The patient should avoid mosquito bites.
2. Avoid high temperature environment: The patient should avoid scalding water; do not apply a hot compress to the affected arm; do not use too high water temperatures when bathing; avoid prolonged hot baths or saunas; and avoid high-temperature environments such as hot sunny days.
3. Avoid weight bearing: The patient should avoid weight bearing (generally not exceeding 500 g) on the upper limbs within 2–4 weeks after surgery. After 4 weeks, slow, gradual increases in muscle and muscular endurance activities, especially resistance training, are required. The patient should avoid heavy physical labor and strenuous physical activities.
4. Avoid compression of proximal upper extremities: The patient should avoid wearing tight clothes, measuring their blood pressure, and lying on the affected side.
5. Pay attention to the sleeping position to ensure the quality of sleep: The patient should sleep in the supine position, and the affected limb should be raised so the hand and arm are in a straight line and the height of the palm is above the level of the heart; or lie on the unaffected side, with the affected limb placed on the side of the body or raised above the level of the heart with pillows. A good sleep can help patients relax, stimulate the vagus nerve, activate the lymphatic system, and prevent and improve lymphedema.
6. Other: Patients should be educated to help them identify edema early and understand its risks. They should be encouraged to restore their arm function as soon as possible; wear preventive elastic cuffs when flying, traveling long distances, or staying at high altitudes; and perform appropriate physical exercise under the guidance of a doctor to avoid excessive fatigue.

Level of evidence: low; recommendation level: recommended

#### Treatment of upper extremity lymphedema

The treatments include conservative treatment and surgical treatment. Conservative treatment refers to comprehensive detumescence therapy, including artificial lymphatic drainage, pressure bandage therapy, skin care, functional exercise, etc., which require the participation of multiple disciplines. Surgical treatment includes lymph node transplantation, lymphatic anastomosis, etc., but its worth has yet to be confirmed by large studies. If symptoms such as redness, swelling, fever, pain, and sudden aggravation of edema appear in the affected arm, the possibility of lymphangitis should be considered, and a hemogram should be done soon and anti-inflammatory treatment given.

Level of evidence: low; recommendation level: recommended

### Concurrent diseases

#### Risk management of cardiovascular and cerebrovascular events

1. Cardiotoxicity management

Underlying cardiac diseases should be fully evaluated to avoid the use of cardiotoxic drugs in this population. Concomitant administration of dexrazoxane with anthracycline therapy can be considered; if cardiac dysfunction is suspected, angiotensin-converting enzyme inhibitors, angiotensin receptor blockers, and specific beta-blockers can be used, as these can help prevent anthracycline-induced cardiomyopathy.

During treatment or the follow-up period after treatment, if cardiac symptoms and signs, an abnormal myocardial enzyme spectrum, or an abnormal cardiac ultrasound is found, the drug should be discontinued promptly and the patient should be reexamined. If the abnormalities persist, the drugs that cause cardiac harm should be discontinued immediately and treatment should be given promptly, and multidisciplinary experts should be invited to participate in the diagnosis and treatment.

(2) Blood lipid management

Lifestyle intervention can help prevent the occurrence of dyslipidemia. The patient’s blood lipids should be tested regularly. Whether to start lipid-lowering drug therapy should be decided based on the patient’s clinical history and/or risk factors. Statins are the most commonly used lipid-lowering drugs in clinical practice and do not interact with endocrine drugs. The simultaneous use of third-generation AIs and statins can not only reduce blood lipids but also prolong DFS in breast cancer patients.

Level of evidence: intermediate; level of recommendation: recommended

#### Fracture risk management

Fracture prevention measures and lifestyle interventions should be taught to all postmenopausal patients and those taking third-generation AIs. In addition to improving lifestyle, patients with intermediate or high risk of fracture should be given appropriate drugs (calcium, vitamin D, bisphosphonates, denosumab, etc.) in a timely manner, and BMD should be closely monitored.

Level of evidence: intermediate; level of recommendation: recommended

### Lifestyle management

A growing body of evidence suggests that the lifestyle of breast cancer patients affects the prognosis. Personal lifestyle-related factors such as diet and nutrition, BMI changes, physical activity, and smoking and drinking after a breast cancer diagnosis are associated with metastasis, relapse, DFS, and mortality. Long-term survival of breast cancer patients requires not only medical and rehabilitation services but also guidance on daily life to help them start and adhere to healthy lifestyle habits, as these can improve their treatment effects, prognosis, quality of life, and survival rate.

#### BMI management

After treatment, breast cancer patients should try to restore their BMI to the normal range, i.e., 18.5-23.9 kg/m^2^, or normal BMI standard in accordance with *The Guidelines for Prevention and Control of Overweight and Obesity in Chinese Adults*.

Level of evidence: low; recommendation level: recommended

#### Nutrition and exercise

The choice of food and the size of each meal (three per day) should be based on the *Chinese food pagoda for a balanced diet*. A diet rich in fruits, vegetables, whole grains, poultry, and fish is recommended, while refined grains, red and processed meats, desserts, high-fat dairy products, and fried potatoes should be reduced.

Breast cancer patients are advised not to smoke, to avoid second-hand smoke, and not to drink alcohol. Regarding health products and dietary supplements, the following are recommended:

1. Try to get the necessary nutrients from the diet as much as possible.
2. Only when clinical or biochemical indicators indicate nutrient deficiency is it necessary to consider taking corresponding nutrient supplements under the guidance of a nutritionist.
3. After a nutritionist deems that the patient cannot get enough nutrients from food and the intake of a nutrient continuously drops to 2/3 of the recommended amount, the patient can consider taking nutrient supplements.

Level of evidence: low; recommendation level: recommended

It is recommended that breast cancer patients avoid a sedentary lifestyle after diagnosis and resume their daily level of physical activity from before the diagnosis as soon as possible. Adults aged 18-64 should do at least 150 minutes of moderate-intensity exercise or 75 minutes of high-intensity aerobic exercise every week, as well as strength training at least twice a week. Patients over 65 years old should try to exercise according to the above guidelines. For those with chronic diseases that limit their movement, the exercise time and intensity should be adjusted according to the doctor’s instructions.

Level of evidence: low; recommendation level: recommended

### Psychological and social support

#### Psychological support

Medical staff can help improve patients’ self-control ability with respect to cognition, decision-making, coping skills, etc., and guide them to appropriately use various coping skills, such as suggestion and catharsis, to increase their tolerance for difficult situations. The importance of maintaining normalcy should be emphasized to help them get out of “patient” mode as soon as possible and face life positively.

1. Provide enough information to help patients rationally accept the reality of having the illness. Medical staff can support the cognitive adjustment of patients to help them engage in appropriate reflection and avoid negative thought patterns and fear.
2. Help patients find a positive purpose to their survival and rebuild their confidence in their life. Medical staff must promptly and correctly assess patients’ current expectations, including dependencies between patients and their families, and encourage them to participate in social activities to recover a normal social life.
3. Stimulate patients’ sense of commitment and help them control themselves effectively. The patient-centered medical care model should be implemented to help patients give full play to their decision-making power and inspire their sense of self-responsibility.

If moderate or severe psychological abnormalities are found in the evaluation, interdisciplinary comprehensive treatment methods, including drug therapy, should be applied, and the patient should be closely followed up.

Level of evidence: low; recommendation level: recommended

#### Social support

Social support networks for breast cancer patients should include professional support, family support, and peer support.

1. Professional support: mainly to provide medical information and psychological support, for which rehabilitation courses, professional lectures, rehabilitation hotlines, rehabilitation office, rehabilitation websites, and rehabilitation-related books can be provided through various new media platforms, mobile applications, etc.
2. Family support: mainly to encourage family members to participate in the patient’s diagnosis, treatment, and rehabilitation processes, for which family information consultation platforms can be provided to facilitate communication between family members.
3. Peer support: mainly to be done by volunteers who are recovered patients, in the form of ward visits or new patient symposiums, which should be conducted under the professional guidance and supervision of medical staff.

Level of evidence: low; recommendation level: recommended

### Sex life and fertility

#### Sex life

A healthy and moderate sex life is conducive to the physical and mental recovery of breast cancer patients. The only thing they need to be reminded of is to always use contraception. The physical barrier contraceptive method is recommended, while hormonal contraceptives should be avoided.

1. The patient should be made aware that the ability to obtain pleasure through touch will not change, regardless of the treatment approach.
2. Patients should be reminded that they can enjoy sexual pleasure in different ways, and partners should help each other and achieve orgasm through touching and caressing.
3. The patient is encouraged to communicate with any sexual partner or consult professionals about sexual issues.

Level of evidence: low; recommendation level: recommended

#### Fertility and fertility preservation

Although there is no evidence that pregnancy harms the prognosis of breast cancer patients, the risk of disease recurrence and the impact of treatment on their offspring must be fully considered when choosing whether and when to conceive, and patients must be fully educated on the topic. Pregnancy may be considered in the following situations:

1. After surgery and radiotherapy in patients with breast carcinoma in situ.
2. Two years after surgery in patients with lymph node–negative invasive breast cancer.
3. Five years after surgery in patients with lymph node–positive invasive breast cancer.
4. For patients who need adjuvant endocrine therapy, the endocrine therapy can be stopped from 3 months before conception until the end of breastfeeding, after which endocrine therapy can be resumed.

Fertility preservation methods should be considered before systemic treatment. Commonly used fertility preservation methods include embryo cryopreservation, egg freezing, and cryopreservation of ovarian tissue. The efficacy of using gonadotropin-releasing hormone analogs to protect ovarian function during chemotherapy still needs to be confirmed by large clinical studies [81].

Level of evidence: low; recommendation level: recommended

## TCM treatment of breast cancer

Breast cancer belongs to the category of “*ruyan*” in TCM and is one of the most common and important cancers that endanger life and health. With the advancements of modern medicine and the continuous emergence of new drugs, the treatment efficiency and survival rate have been significantly improved. TCM has occupied a certain position in the comprehensive treatment of breast cancer. TCM can improve the patient’s symptoms; synergistically improve postoperative recovery; reduce adverse reactions to radiotherapy, chemotherapy, endocrine therapy, molecular-targeted therapy, and molecular immunotherapy while increasing the efficacy of various therapies; regulate the patient’s immune function and physical status; prevent and treat tumors and complications related to the tumor treatment; prevent relapse and metastasis; improve the quality of life; and possibly prolong the patient’s survival. Thus, TCM represents an important auxiliary method of breast cancer treatment [82].

According to the pathogenesis and characteristics of breast cancer, combined with the method of syndrome differentiation and treatment of metastasis and the method of diagnosing and treating according to the individual’s condition, TCM clinically advocates treatment through clinical stage–based syndrome differentiation. That is, in the perioperative period, perichemotherapy period, periradiotherapy period, and consolidation (rehabilitation) period, the general treatment principles of *fuzhen* (strengthening vital *qi*) and *quxie* (dispelling pathogenic factors) are adopted and cover the entire course of breast cancer treatment.

There are clear guidelines for selecting the suitable population for TCM treatment of breast cancer. For patients with access to Western medicine treatment conditions, Western medicine treatment should be adopted as the main treatment method, while TCM can be a supplementary treatment. For patients who are not suited for or are unwilling to accept Western medicines, TCM alone can be used. TCM treatment is a useful supplement in the clinical tumor-free rehabilitation period and the advanced-cancer palliative care period [82–85].

The current clinical methods of TCM for breast cancer mainly include TCM decoctions and granules, patent prescriptions, injections, and external preparations as well as nondrug treatments (e.g., *qigong*, acupuncture). TCM decoctions play a dominant role and can be used in the syndrome differentiation treatment of breast cancer symptoms and related posttreatment conditions according to the patient’s specific circumstances. The so-called folk prescriptions or empirical formulas need to be viewed with caution [82, 83].

For breast cancer, TCM emphasizes the idea of preventing the disease before its onset, i.e., prevention first and, once the disease occurs, preventing its exacerbation. For some subhealthy or high-risk individuals (including those with breast tumors), TCM also uses decoctions and proprietary medicines (e.g., Xiaojinwan, Xihuangwan, etc.) in clinical treatment. As research progresses, the category of evidence is expected to increase [82–85].

In addition, several tasks are involved in the nondrug treatment of breast diseases by TCM: First, TCM has always paid attention to the emotional recuperation of breast cancer patients to improve their psychological endurance and physical and mental state, which greatly helps the patient’s clinical recovery. Second, appropriate functional exercises (e.g., Tai Chi, yoga, Wu Qin Xi (five-animal Qigong exercises), etc.) can be integrated to help in the recovery. Third, given that the onset of breast cancer itself is related to diet, a reasonable diet not only supplies the necessary nutrients but also is a part of treatment, and part of TCM is its diet therapy.

It is worth mentioning that in the process of breast cancer treatment, we should believe in the efficacy of TCM while being not superstitious about it.

Level of evidence: low; recommendation level: recommended

## Appendix

Tables 8, 9, 10 and 11

**Table 8:** Expert consensus on recommending *BRCA* genetic testing for breast cancer patients

Expert consensus on recommending *BRCA* genetic testing for breast cancer patients	
•Has known deleterious mutations of the *BRCA1*/*2* gene in the family •History of breast cancer meeting the following criteria: Age at diagnosis ≤ 45 years Age at diagnosis: 46-50 years ▶ Second primary breast cancer ▶ ≥ 1 immediate family member diagnosed with breast cancer, regardless of age at diagnosis ▶ ≥ 1 immediate family member diagnosed with high-grade prostate cancer (Gleason score ≥ 7) ▶ Limited or unknown family history of triple-negative breast cancer diagnosed at age ≤ 60 regardless of age, but meeting any of the following criterion: ▶ ≥ 1 immediate family member meeting one of the following criteria: Age ≤ 50 at diagnosis of breast cancer or ovarian cancer, or male breast cancer, or metastatic prostate cancer, or pancreatic cancer ▶ ≥ 2 patients or immediate family members diagnosed with breast cancer •History of ovarian cancer •History of male breast cancer •History of pancreatic cancer •History of metastatic prostate cancer •History of high-grade prostate cancer (Gleason score ≥7) regardless of age and meeting one of the following criteria: ▶ ≥1 immediate family member diagnosed with ovarian cancer, pancreatic cancer, or metastatic prostate cancer regardless of age at diagnosis or with breast cancer at age ≤ 50 ▶ ≥2 immediate family members diagnosed with breast cancer or prostate cancer (any grade), regardless of age at diagnosis •*BRCA1*/*2* deleterious mutations found in the tumor and with unknown germline mutation status •Regardless of family history, *BRCA* mutation–associated cancers benefit from targeted therapy (e.g., PARP inhibitor therapy for ovarian cancer/metastatic HER2-negative breast cancer, platinum-based chemotherapy for prostate cancer) •Individuals who do not meet the above criteria but have ≥ 1 first- or second-degree relative who meets any of the above criteria. Interpretation of test results for noncarriers (*BRCA1*/*2* deleterious mutations) has limitations and needs to be fully discussed	

Note: Further risk assessment, genetic counseling, and genetic testing and management should be considered if one or more of the above criteria are met. Genetic test results of individuals with only a family history should be interpreted with caution, as the results may have significant limitations

**Table 9:** VNPI

VNPI	
VNPI = A + B + C + D	
A = tumor size	
1: ≤ 15 mm	
2:16–40 mm	
3: ≥ 41 mm	
B = surgical margin condition	
1: ≥ 10 mm	
2:1–9 mm	
3: <1 mm	
C = nuclear grade	
1: Low grade	
2: Intermediate grade	
3: Advanced grade	
D = age	
1: ≥ 60 years old	
2 :40–59 years old	
3: < 40 years old	

**Table 10:** Commonly used adjuvant/neoadjuvant treatment options for breast cancer^a^

1 Adjuvant/neoadjuvant therapy for HER2-negative breast cancer	
TAC regimen	
Docetaxel 75 mg/m^2^ iv on day 1	
Doxorubicin 50 mg/m^2^ iv on day 1	
Cyclophosphamide 500 mg/m^2^ iv on day 1	
21 d/cycle, 6 cycles in total	
(All cycles are supported with G-CSF/PEG-rhG-CSF)	
Dose-intensive AC/EC→P (once every 2 weeks)	
Doxorubicin 60 mg/m^2^ iv on day 1	
or epirubicin 90–100 mg/m^2^ iv on day 1	
Cyclophosphamide 600 mg/m^2^ iv on day 1	
14 d/cycle, 4 cycles in total	
Sequentially with	
Paclitaxel 175 mg/m^2^ iv 3 h on day 1:14 d/cycle, 4 cycles in total	
(All cycles are supported with G-CSF/PEG-rhG-CSF)	
Dose-dense AC/EC→P (once every week)	
Doxorubicin 60 mg/m^2^ iv on day 1	
or epirubicin 90–100 mg/m^2^ iv on day 1	
Cyclophosphamide 600 mg/m^2^ iv on day 1	
14 d/cycle, 4 cycles in total (supported with G-CSF/PEG-rhG-CSF)	
Sequentially with	
Paclitaxel 80 mg/m^2^ iv 1 h on day 1: once a week, 12 weeks in total	
AC/EC→P/T regimen	
Doxorubicin 60 mg/m^2^ iv on day 1	
or epirubicin 90–100 mg/m^2^ iv on day 1	
Cyclophosphamide 600 mg/m^2^ iv on day 1	
21 d/cycle, 4 cycles in total	
Sequentially with	
Paclitaxel 80 mg/m^2^ iv 1 h on day 1: once a week, 12 weeks in total	
Or docetaxel 100 mg/m^2^ iv on day 1:21 d/cycle, 4 cycles in total	
TC regimen (for adjuvant therapy)	
Docetaxel 75 mg/m^2^ iv on day 1	
Cyclophosphamide 600 mg/m^2^ iv on day 1	
21 d/cycle, 4–6 cycles in total	
AC regimen	
Doxorubicin 60 mg/m^2^ iv on day 1	
Cyclophosphamide 600 mg/m^2^ iv on day 1	
21 d/cycle, 4 cycles in total	
EC regimen	
Epirubicin 100 mg/m^2^ iv on day 1	
Cyclophosphamide 830 mg/m^2^ iv on day 1	
21 d/cycle, 4 cycles in total	
PCb regimen	
Paclitaxel 80 mg/m^2^, on days 1, 8, 15	
Carboplatin area under curve (AUC) = 6 on day 1, or AUC = 2 on days 1, 8, 15	
21 d/cycle, 4–6 cycles in total	
TCb regimen	
Docetaxel 75 mg/m^2^ on day 1	
Carboplatin AUC = 6 on day 1	
21 d/cycle, 4–6 cycles in total	
Intensive adjuvant therapy	
[1] XT→XEC regimen (for triple-negative breast cancer)	
Docetaxel 75 mg/m^2^ iv on day 1	
Capecitabine 1,000 mg/m^2^ po bid on days 1-14	
21 d/cycle, 4 cycles in total	
Sequentially with	
Epirubicin 75 mg/m^2^ iv on day 1	
Cyclophosphamide 600 mg/m^2^ iv on day 1	
Capecitabine 1,000 mg/m^2^ po bid on days 1-14	
21 d/cycle, 4 cycles in total	
[2] Dose-intensive X after standard chemotherapy (for triple-negative breast cancer)	
Capecitabine 650 mg/m^2^ po bid, orally for 1 year continuously	
[3] Dose-intensive X after neoadjuvant does not reach pCR (for triple-negative breast cancer and lymph node residual–positive ER-positive/HER2-negative breast cancer)	
Capecitabine 1,250 mg/m^2^ po bid, on days 1-14, for a total of 8 cycles	
[4] Olaparib enhancement (for pathogenic/suspected pathogenic g*BRCA* mutation high-risk breast cancer that does not reach indication for adjuvant therapy yet)	
Olaparib 300 mg po bid, orally for 1 year	
Neoadjuvant endocrine therapy for ER-positive/HER2-negative patients: postmenopausal patients usually use AI for neoadjuvant endocrine therapy; premenopausal patients should not be routinely subjected to neoadjuvant endocrine therapy unless they enter a clinical study or have contraindications to chemotherapy (OFS+AI/fulvestrant is optional).	
2. Adjuvant/neoadjuvant therapy for HER2-positive breast cancer	
AC/EC→PH	
Doxorubicin 60 mg/m^2^ iv on day 1	
or epirubicin 90–100 mg/m^2^ iv on day 1	
Cyclophosphamide 600 mg/m^2^ iv on day 1	
21 d/cycle, 4 cycles in total	
Sequentially with	
Paclitaxel 80 mg/m^2^ iv 1 h day 1	
Trastuzumab 2 mg/kg (with a first dose of 4 mg/kg) on day 1	
Once a week, for a total of 21 weeks	
Then trastuzumab 6 mg/kg, once every 3 weeks for 1 year	
Monitor cardiac function once every 3 months	
Dose-dense AC/EC→PH regimen	
Doxorubicin 60 mg/m^2^ iv on day 1	
or epirubicin 90–100 mg/m^2^ iv on day 1	
Cyclophosphamide 600 mg/m^2^ iv on day 1	
14 d/cycle, 4 cycles in total	
Sequentially with	
Paclitaxel 175 mg/m^2^ iv 3 h on day 1, 14 d/cycle, 4 cycles in total	
(All cycles are supported with G-CSF/PEG-rhG-CSF)	
At the same time, trastuzumab is given as a first dose of 4 mg/kg and then 2 mg/kg once a week for 1 year	
Trastuzumab can also be given after the end of paclitaxel, with a first dose of 8 mg/kg followed by 6 mg/kg once every 3 weeks for 1 year.	
Cardiac function should be monitored at baseline and months 3, 6, and 9	
AC/EC→TH regimen	
Doxorubicin 60 mg/m^2^ iv on day 1	
or epirubicin 90–100 mg/m^2^ iv on day 1	
Cyclophosphamide 600 mg/m^2^ iv on day 1	
21 d/cycle, 4 cycles in total	
Sequentially with	
Docetaxel 100 mg/m^2^ iv on day 1	
Trastuzumab 2 mg/kg (with a first dose of 4 mg/kg) on days 1, 8, and 15,	
21 d/cycle, 4 cycles in total, and then trastuzumab at 6 mg/kg once every 3 weeks for 1 year	
Cardiac function should be examined once every 3 months	
TCbH regimen	
Docetaxel 75 mg/m^2^ iv on day 1	
Carboplatin AUC =6 iv on day 1	
Trastuzumab 6 mg/kg (with a first dose of 8 mg/kg) on day 1	
21 d/cycle, 6 cycles in total	
Then trastuzumab 6 mg/kg once every 3 weeks for 1 year	
Cardiac function should be examined once every 3 months	
AC/EC→THP regimen	
Doxorubicin 60 mg/m^2^ iv on day 1	
or epirubicin 90–100 mg/m^2^ iv on day 1	
Cyclophosphamide 600 mg/m^2^ iv on day 1	
21 d/cycle, 4 cycles in total	
Sequentially with	
Docetaxel 75-100 mg/m^2^ iv on day 1	
or paclitaxel 80 mg/m^2^ iv 1 h on days 1, 8, and 15	
Trastuzumab 6 mg/kg (with a first dose of 8 mg/kg) on day 1	
Pertuzumab 420 mg iv (with a first dose of 840 mg) on day 1	
21 d/cycle, 4 cycles in total	
Then trastuzumab 6 mg/kg + pertuzumab 420 mg once every 3 weeks for 1 year	
Cardiac function should be examined once every 3 months	
Dose-dense AC/EC→THP regimen	
Doxorubicin 60 mg/m^2^ iv on day 1	
or epirubicin 90–100 mg/m^2^ iv on day 1	
Cyclophosphamide 600 mg/m^2^ iv on day 1	
14 d/cycle, 4 cycles in total (supported by G-CSF/PEG-rhG-CSF)	
Sequentially with	
Docetaxel 75-100 mg/m^2^ iv on day 1	
or paclitaxel 80 mg/m^2^ iv 1 h on days 1, 8, and 15	
Trastuzumab 6 mg/kg (with a first dose of 8 mg/kg) day 1	
Pertuzumab 420 mg (with a first dose of 840 mg) iv on day 1	
21 d/cycle, 4 cycles in total	
Then trastuzumab 6 mg/kg + pertuzumab 420 mg once every 3 weeks for 1 year	
Cardiac function should be examined once every 3 months	
TCbHP regimen	
Docetaxel 75 mg/m^2^ iv on day 1	
Carboplatin AUC =6 iv on day 1	
Trastuzumab 6 mg/kg (with a first dose of 8 mg/kg) day 1	
Pertuzumab 420 mg (with a first dose of 840 mg) iv on day 1	
21 d/cycle, 6 cycles in total	
Then trastuzumab 6 mg/kg + pertuzumab 420 mg once every 3 weeks for 1 year	
Cardiac function should be examined once every 3 months	
wTH regimen (for adjuvant therapy)	
Paclitaxel 80 mg/m^2^ iv 1 h day 1	
Trastuzumab 2 mg/kg (with a first dose of 4 mg/kg) iv on day 1	
Once a week, for a total of 12 weeks	
Then trastuzumab 6 mg/kg once every 3 weeks for 1 year	
Cardiac function should be examined once every 3 months	
TC+H regimen (for adjuvant therapy)	
Docetaxel 75 mg/m^2^ iv on day 1	
Cyclophosphamide 600 mg/m^2^ iv on day 1	
Trastuzumab 6 mg/kg (with a first dose of 8 mg/kg) day 1	
21 d/cycle, 4 cycles in total	
Then followed by trastuzumab 6 mg/kg once every 3 weeks for 1 year	
Cardiac function should be examined once every 3 months	
Adjuvant intensive therapy regimen (for high-risk HER-positive breast cancer, especially ER+ patients)	
Neratinib 240 mg po qd for one year after completion of trastuzumab-containing therapy	

^a^ In the above adjuvant therapy, nab-paclitaxel can be tried to replace paclitaxel or docetaxel when medically necessary (e.g., reducing allergic reactions, etc.), but with a weekly therapeutic dose not exceeding 125 mg/m^2^

**Table 11:** Commonly used chemotherapy and targeted therapy regimens for recurrent or metastatic breast cancer

1. Chemotherapy and targeted therapy regimens commonly used in HER2-negative breast cancer	
[1] Monotherapy	
Anthracyclines	
Doxorubicin 60–75 mg/m^2^ iv on day 1	
21 d/cycle	
or doxorubicin 20 mg/m^2^ iv once a week	
Epirubicin 60–90 mg/m^2^ iv on day 1	
21 d/cycle	
Liposomal doxorubicin 50 mg/m^2^ iv on day 1	
28 d/cycle	
Taxanes	
Paclitaxel 175 mg/m^2^ iv on day 1	
21 d/cycle	
or paclitaxel 80 mg/m^2^ iv once a week	
Docetaxel 60-100 mg/m^2^ iv on day 1	
21 d/cycle	
Nab-paclitaxel 100–150 mg/m^2^ iv on days 1, 8, and 15	
28 d/cycle	
or nab-paclitaxel 260 mg/m^2^ iv on day 1	
21 d/cycle	
Antimetabolites	
Capecitabine 1,000-1,250 mg/m^2^ po bid on days 1-14	
21 d/cycle	
Gemcitabine 800–1 200 mg/m^2^ iv on days 1, 8, and 15	
28 d/cycle	
Other microtubule inhibitors	
Vinorelbine 25 mg/m^2^ iv once a week or 50 mg po on days 1, 8, and 15	
Eribulin 1.4 mg/m^2^ iv on days 1 and 8	
21 d/cycle	
Utidelone 30 mg/m^2^ iv on days 1-5 for a 21-d cycle	
Platinum-based drugs (for triple-negative breast cancer or breast cancer with known *BRCA1*/*2* mutations)	
Cisplatin 75 mg/m^2^ iv on day 1 or 25 mg/m^2^ iv on days 1-3	
21 d/cycle	
Carboplatin AUC = 5–6 iv on day 1	
21–28 d/cycle	
PARP inhibitor (for breast cancer with known *BRCA1*/*2* mutations; has not obtained indications in China yet)	
Olaparib 300 mg po bid	
Anti-TROP2 ADC (for triple-negative breast cancer; not yet approved for marketing in China)	
Sacituzumab govitecan-hziy 10 mg/kg iv on days 1 and 8	
21 d/cycle	
[2] Combination therapy	
XT regimen	
Docetaxel 75 mg/m^2^ iv on day 1	
or nab-paclitaxel 100-150 mg/m^2^ iv on day 1	
Once a week	
Capecitabine 1,000 mg/m^2^ po bid on days 1-14	
21 d/cycle	
GT regimen	
Paclitaxel 175 mg/m^2^ iv on day 1	
Gemcitabine 1,000–1,250 mg/m^2^ iv on days 1 and 8	
21 d/cycle	
NX regimen	
Vinorelbine 25 mg/m^2^ iv on days 1 and 8 or 40 mg po on days 1, 8, and 15	
Capecitabine 1,000 mg/m^2^ po bid on days 1-14	
21 d/cycle	
GP regimen (for triple-negative breast cancer)	
Gemcitabine 1,000–1,250 mg/m^2^ iv on days 1 and 8	
Cisplatin 75 mg/m^2^ iv on day 1 or 25 mg/m^2^ iv on days 1-3	
21 d/cycle	
GC regimen (for triple-negative breast cancer)	
Gemcitabine 1,000 mg/m^2^ iv on days 1 and 8	
Carboplatin AUC = 2 iv on days 1 and 8	
21 d/cycle	
AP regimen (for triple-negative breast cancer)	
Nab-paclitaxel 125 mg/m^2^ iv on days 1 and 8	
Cisplatin 75 mg/m^2^ iv on day 1 or 25 mg/m^2^ iv on days 1-3	
21 d/cycle	
NP regimen (for triple-negative breast cancer)	
Vinorelbine 25 mg/m^2^ iv on days 1 and 8	
Cisplatin 75 mg/m^2^ iv on day 1 or 25 mg/m^2^ iv on days 1-3	
Or carboplatin AUC = 2 iv on days 1 and 8	
21 d/cycle	
PC regimen	
Paclitaxel 175 mg/m^2^ iv on day 1	
or nab-paclitaxel 125 mg/m^2^ iv on days 1 and 8	
Carboplatin AUC = 5–6 day 1, or AUC = 2 iv on days 1 and 8	
21 d/cycle	
Paclitaxel + bevacizumab (bevacizumab has not yet been approved for indications in China)	
Paclitaxel 90 mg/m^2^ iv on day 1, 8, and 15	
Bevacizumab 10 mg/kg days 1 and 15	
28 d/cycle	
Immunotherapy regimens containing PD-1/PD-L1 antibody (can be used for triple-negative breast cancer, but their indications have not yet been approved in China)	
① Atezolizumab + nab-paclitaxel (when PD-L1 SP142 is positive, i.e., IC ≥ 1%)	
Atezolizumab 840 mg iv on days 1, 15	
Nab-paclitaxel 100 mg/m^2^ iv on days 1, 8, and 15	
28 d/cycle	
② Pembrolizumab + chemotherapy (when PD-L1 22C3 CPS ≥ 10)	
Pembrolizumab 200 mg iv on day 1, 21 d/cycle	
Nab-paclitaxel 100 mg/m^2^ iv on days 1, 8, and 15	
28 d/cycle	
Or paclitaxel 90 mg/m^2^ iv on days 1, 8, and 15; 28 d/cycle	
Or gemcitabine 1,000 mg/m^2^ iv on the 1st day + carboplatin AUC = 2 iv on days 1 and 8; 21 d/cycle	
2. Chemotherapy and targeted therapy regimens commonly used in HER2-positive breast cancer	
THP regimen	
Docetaxel 75 mg/m^2^ iv on day 1	
Or nab-paclitaxel 100-150 mg/m^2^ iv once a week on day 1	
or paclitaxel 80 mg/m^2^ iv once a week on day 1	
Trastuzumab first dose of 8 mg/kg followed by 6 mg/kg iv on day 1	
Pertuzumab first dose 840 mg, followed by 420 mg iv on day 1	
21 d/cycle	
TXH regimen	
Docetaxel 75 mg/m^2^ iv on day 1	
Capecitabine 1,000 mg/m^2^ po bid on days 1-14	
Trastuzumab (with a first dose of 8 mg/kg) followed by 6 mg/kg iv on day 1	
21 d/cycle	
TH regimen	
Nab-paclitaxel 100-150 mg/m^2^ iv on day 1	
Trastuzumab (with a first dose of 4 mg/kg) followed by 2 mg/kg iv on day 1	
7 d/cycle	
or	
Docetaxel 75 mg/m^2^ iv on day 1	
Trastuzumab (with a first dose of 8 mg/kg) followed by 6 mg/kg iv on day 1	
21 d/cycle	
NH Regimen	
Vinorelbine 30 mg/m^2^ iv on days 1 and 8	
Trastuzumab (with a first dose of 4 mg/kg) followed by 2 mg/kg iv on day 1	
21d/cycle	
or	
Vinorelbine 25 mg/m^2^ iv on days 1, 8, and 15	
Trastuzumab or inetetamab (with a first dose of 4 mg/kg) followed by 2 mg/kg iv on day 1	
28 d/cycle	
XH regimen	
Capecitabine 1,000-1,250 mg/m^2^ po bid on days 1-14	
Trastuzumab (with a first dose of 8 mg/kg) followed by 6 mg/kg iv on day 1	
21 d/cycle	
PCbH	
Paclitaxel 175 mg/m^2^ iv on day 1	
or nab-paclitaxel 125 mg/m^2^ iv on days 1 and 8	
Carboplatin AUC = 5–6 day 1, or AUC = 2 iv on days 1 and 8	
Trastuzumab (with a first dose of 8 mg/kg) followed by 6 mg/kg iv on day 1	
21 d/cycle	
Pyrotinib + capecitabine regimen	
Pyrotinib 400 mg po qd	
Capecitabine 1,000 mg/m^2^ po bid on days 1-14	
21 d/cycle	
Neratinib + capecitabine regimen	
Neratinib 240 mg po qd on days 1-21	
Capecitabine 750 mg/m^2^ po bid on days 1-14	
21 d/cycle	
Lapatinib + capecitabine	
Lapatinib 1,250 mg po qd	
Capecitabine 1,000 mg/m^2^ po bid on days 1-14	
21 d/cycle	
Lapatinib + Trastuzumab	
Lapatinib 1,000 mg po qd	
Trastuzumab (with a first dose of 8 mg/kg) followed by 6 mg/kg iv on day 1	
21 d/cycle	
T-DM1 monotherapy	
3.6 mg/kg iv on day 1	
21 d/cycle	
DS8201 (has not been approved for use in China yet)	
5.4 mg/kg iv on day 1	
21 d/cycle	
